# Minimizing wildlife impacts for offshore wind energy development: Winning tradeoffs for seabirds in space and cetaceans in time

**DOI:** 10.1371/journal.pone.0215722

**Published:** 2019-05-14

**Authors:** Benjamin D. Best, Patrick N. Halpin

**Affiliations:** 1 EcoQuants LLC, Santa Barbara, California, United States of America; 2 Marine Geospatial Ecology Laboratory, Nicholas School of the Environment, Duke University, Durham, North Carolina, United States of America; Sichuan University, CHINA

## Abstract

Although offshore wind energy development (OWED) offers a much-needed renewable energy alternative to fossil fuels, holistic and effective methods for evaluating environmental impacts on wildlife in both space and time have been lacking. The lengthy environmental compliance process, estimated to incur a 7–10 year permitting timeline [1], has been identified as a significant impediment to offshore energy development in U.S. waters. During operation, seabirds can collide and be displaced by turbines. During episodic pre-operation phases, cetaceans are most heavily impacted acoustically by pile driving (and similarly seismic air gun surveys for oil and gas exploration). The varying nature of impacts in space and time leads us to conclude that sites should be selected in space to minimize long-term operational impacts on seabirds, and timing of surveying and construction activities to be conducted in times of the year when sensitive migratory marine mammals are least present. We developed a novel spatiotemporal decision support framework that interactively visualizes tradeoffs between OWED industry profits and wildlife sensitivities, in both space and time. The framework highlights sites on a map that are the most profitable and least sensitive to seabirds. Within the U.S. Mid-Atlantic study area, the New York Call Areas are particularly well optimized for minimal impact on seabirds with maximal profits to OWED. For a given site, pre-operational activities (e.g. pile driving and seismic air gun surveying) are advised by cetacean sensitivity across months of the year that minimize impacts on migratory cetaceans, particularly those of highest conservation concern such as the North Atlantic right whale (*Eubalaena Glacialis*). For instance, within optimal sites for the New York Call Area the least impacting months are May and June. Other taxa are certainly affected by OWED and should be incorporated into this framework, but data on their distributions and/or sensitivities is currently less well known. Built with open-source software made publicly available, the authors hope this framework will be extended even more comprehensively into the future as our knowledge on species distributions and OWED sensitivities expands for streamlining environmental compliance.

## Introduction

As of the end of 2017, the total installed offshore wind capacity is at 18,814 megawatts (MW) worldwide with the United Kingdom leading and Germany following at 6,836 MW and 5,355 MW respectively [2]. Europe accounts for 84% of installed capacity with most of the remainder in Asia. The United States presently has just one grid connected production facility in Block Island, RI with a 30 MW capacity. However as of June 2018, the U.S. does have a total project pipeline of 25,434 MW (3,892 MW of project-specific capacity and 21,542 MW of undeveloped lease area potential capacity), according the U.S. Department of Energy. Although other projects are slated for the future, what accounts for this stark lack of development in U.S. waters?

The environmental compliance process, estimated to incur a 7–10 year permitting timeline, has been identified as a significant impediment to offshore energy development in U.S. waters [1]. A “Smart from the Start” interagency program led by the federal leasing agency, the Bureau of Ocean Energy Management (BOEM), has formed task forces to reduce these demands by identifying environmentally responsible Wind Energy Areas (WEAs) for offshore wind development [3]. These areas, however, were the result of many negotiations between a wide variety of stakeholders and not necessarily the result of a systematic, transparent, quantitative process.

Renewable energy development has seen more success on land in the US, which reached 254 gigawatts (GW) of wind capacity and 53 GW of solar at utility scale by 2017 [4]. Lessons on land may translate to improving the efficiency of marine spatial planning [5]. For instance, multi-criteria decision analysis (Stoms et al. 2013) enabled the Bureau of Land Management (BLM) to fast track certain areas for permitting as part of its Desert Renewable Energy Conservation Plan (DRECP) in the southwest US, which were deemed likely to have the least impact on wildlife while providing sufficient wind or solar energy and nearby transmission capabilities to be profitable for development. Almost no human development is without some environmental impact, which are often difficult to quantify. Still, providing this high-level view can flag potential conflict areas where greater caution should be exercised and conversely expedite permitting of other areas, for instance where species of concern are less likely to occur.

The regulatory landscape for environmental compliance and offshore wind permitting in the United States is quite vast, requiring interagency oversight across a broad sweep of regulations [1]. The Energy Policy Act of 2005, an amendment to Outer Continental Shelf Lands Act later, grants BOEM as the lead management authority for offshore wind energy projects in federal waters, which are beyond the 3 nm state waters, except within the national marine sanctuaries and monuments where NOAA is the authority under the National Marine Sanctuaries Act. Wildlife, and in particular endangered species, are given protections under the National Environmental Policy Act, Endangered Species Act, Marine Mammal Protection Act (MMPA), Migratory Bird Treaty Act, and the Bald and Golden Eagle Protection Act. The MMPA protects marine mammals and requires extra authorization through NOAA Fisheries Office of Protected Resources for any incidental harm, particularly for acoustic damage from activities such as pile driving. These federal statutes are considered throughout the process that BOEM oversees, including submission of an initial Site Assessment Plan and subsequent Construction and Operation Plan. The Coastal Zone Management Act encourages consistent protections between federal and state waters.

To make necessary information available to developers BOEM has also been facilitating the input of relevant spatial data into the online MarineCadastre.gov portal. Datasets detail individual species distributions and potential conflicts with other industries, such as military and shipping. Similar portals have been created at a regional level, such as for Mid-Atlantic Regional Council on the Ocean (https://portal.midatlanticocean.org). While the availability of these datasets will no doubt aid the planning process for offshore wind energy development (OWED), a comprehensive summary view of overall risk to wildlife that combines the many datasets is still lacking.

The contrasting tradeoffs between wildlife conservation and energy development can be explicitly modeled in terms of an efficiency frontier [6]. Originally developed as portfolio analysis to weigh financial investment in terms of risk versus return over time (Markowitz 1952), tradeoff analysis provides a useful synoptic view for evaluating across many sites the risk to wildlife versus the profitable return to industry. Ideally, alternative sites can be chosen that maintain profitability while also maximizing conservation benefit. Plotting the value of each site along two axes (i.e. profitability versus conservation) readily yields a relationship, which for the ideal scenario of interacting services is concave across the range of values [7].

Although White et al. [6] explicitly mapped and plotted tradeoffs between whale watching conservation versus wind energy profitability, each scenario was an alternate wind farm configuration. The study was fine in spatial scale and not framed so as to offer spatial preference of one site versus another. Winiarski et al. [8] did offer irreplaceability by site using a Marxan spatial prioritization software from density surface models of birds but did not account explicitly for sensitivity of birds to OWED.

A holistic framework for quantifying sensitivity of birds to OWED was first developed by Garthe & Hüppop [9] to account for species-specific responses to OWED according to direct (collision) and indirect (displacement) effects. This framework has been expanded upon by Furness et al. [10] and explicitly mapped from density surface models in the UK by sensitivity to collision and displacement [11]. A subsequent study [12] incorporated uncertainty to arrive at broadly similar measures of vulnerability.

But how then are other species incorporated to the decision-making process? Goodale & Milman [13] summarize impacts on wildlife in terms of a hazard-vulnerability-exposure model. OWED hazards are considered in terms of: 1) hazard intensity and phases of development (pre-construction, construction, operation, and decommissioning); vulnerability of species; and exposure in terms of space and time; all to be considered cumulatively. Impacts can be both direct, i.e. cause mortality, and indirect, i.e. influence individual behavior so as to reduce reproductive success. Direct impacts of birds and bats colliding with turbines have been reasonably well characterized [13–15] while indirect effects of acoustic disturbance to marine mammals during pile driving has been difficult to quantify [16].

The majority of direct OWED impacts to cetaceans are acoustic, not during operation but with pile driving during construction [16,17]. Both of these activities impart a large amount of acoustic energy that can kill or harm animals in the immediate vicinity [18]. For instance, disturbance of harbor porpoises in Germany was demonstrated to reach distances more than 25 km from the pile driving site [19].

**Fig 1:**
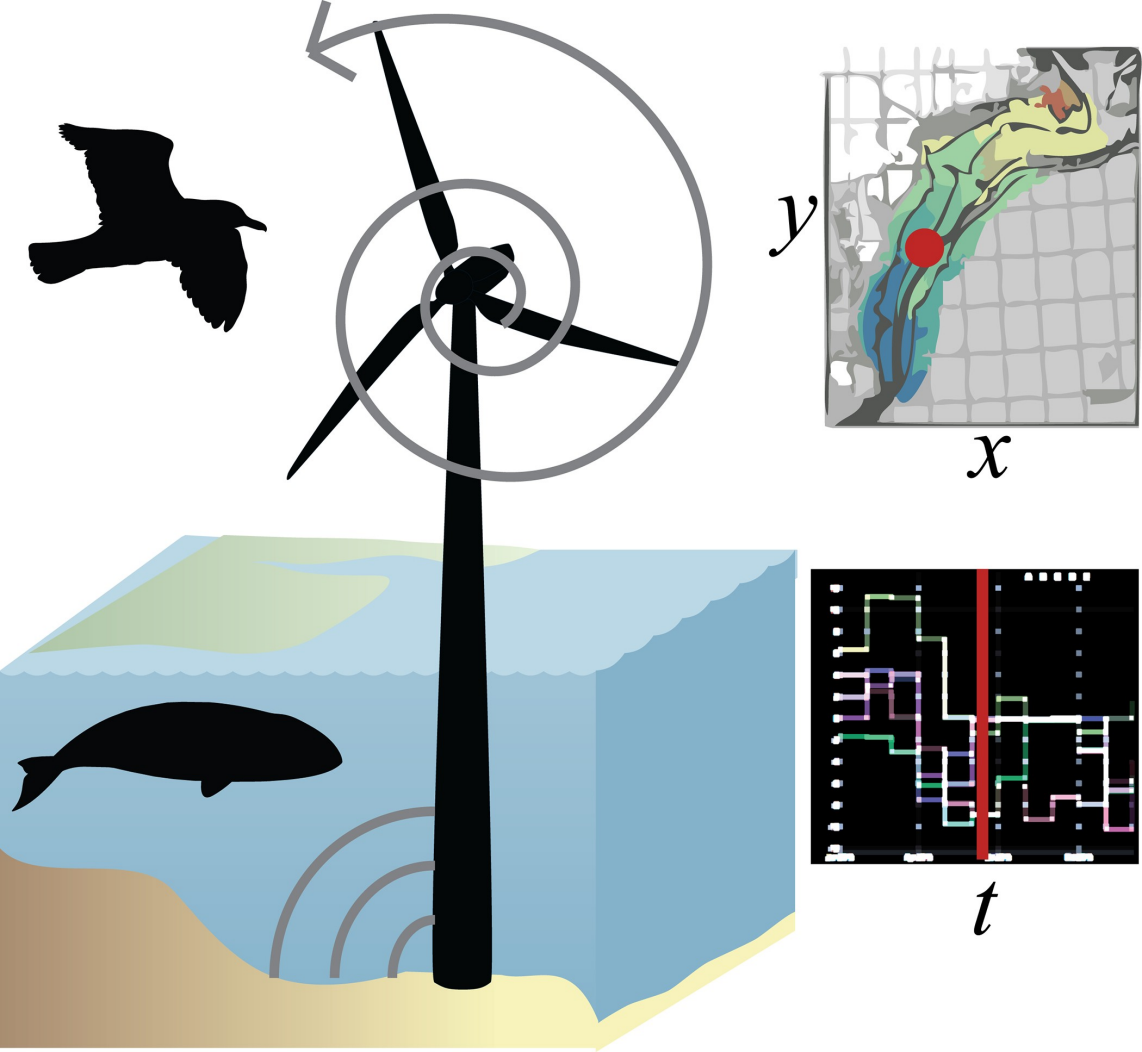
Summary diagram of spatiotemporal tradeoff framework. Since turbines from offshore wind operationally impact seabirds, preferred sites in space (x, y) maximize profitability to wind industry and minimize sensitvity to seabirds. Cetaceans, on the other hand, are mostly impacted episodically by pre-operational activities such as pile driving that impart potentially damaging acoustic energy, so should be timed (t) when species of conservation concern are least present at the given site based on migratory patterns.

In contrast to Europe where marine mammals are mostly resident, the US North Atlantic seaboard has more migratory marine mammals. Effects on birds generally occur during the long-term operation of wind turbines, whereas impacts on marine mammals are most experienced episodically and acoustically during construction. The varying nature of impacts in space and time leads us to conclude that sites should be selected in space to minimize long-term impacts on birds, and timing of surveying and construction activities to be conducted in times of the year when sensitive migratory marine mammals are least present (Fig 1). The goal of this study is to describe an interactive decision support framework that explores the economic and environmental tradeoffs in space and time to find optimal sites that minimize impact to wildlife while preserving profitability to OWED, using the US Mid-Atlantic as a case study area.

## Methods

**Fig 2:**
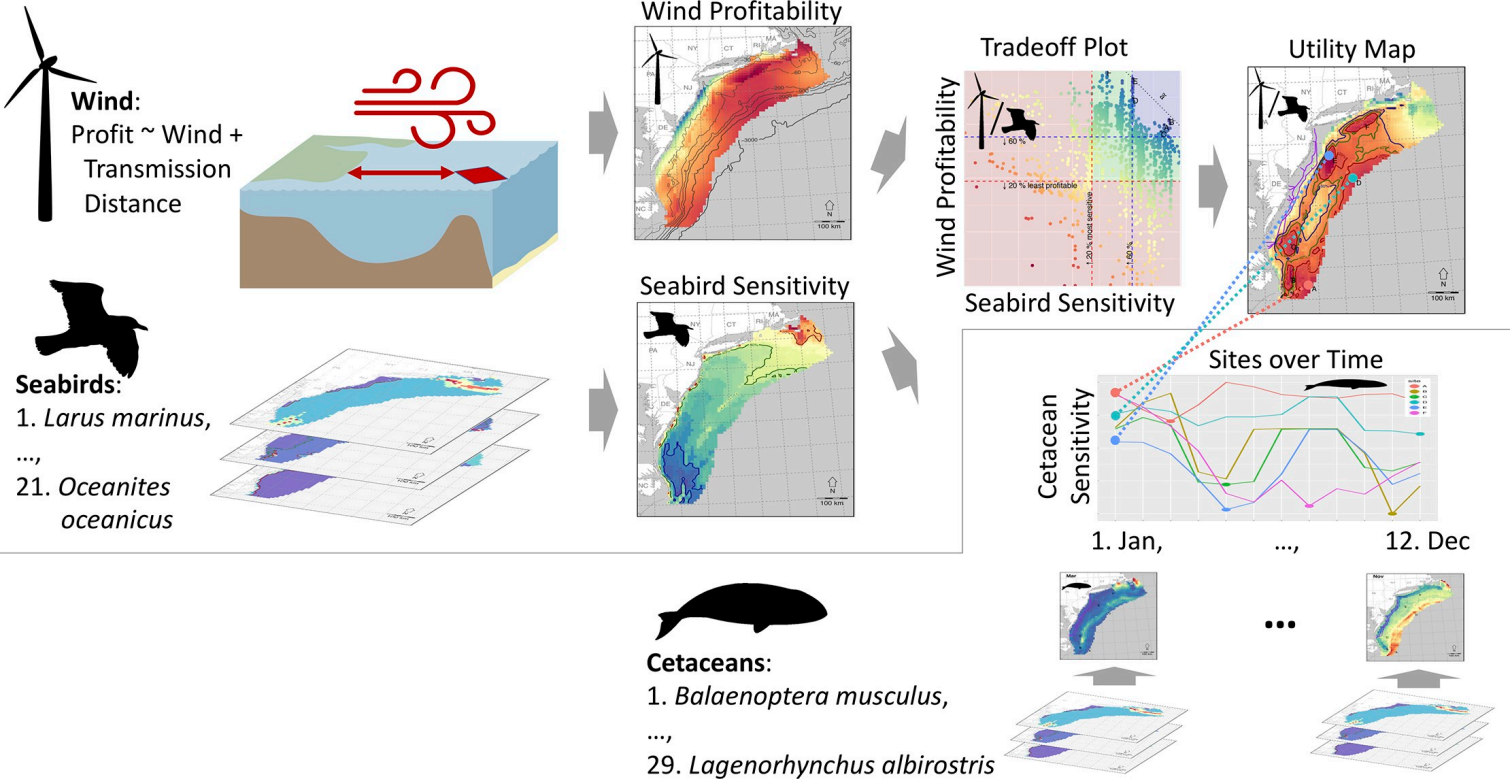
Overview of methods for bringing together wind profitability, seabird sensitivity over space, and cetacean sensitivity over space and time. (See text for more detailed description).

To realize the general concept of the spatiotemporal framework (Fig 1) three components must be analyzed and brought together (Fig 2): 1) offshore wind energy profitability over space, 2) seabird sensitivity over space, and 3) cetacean sensitivity over space and time. Profitability to the wind industry is estimated as a function of transmission distance and wind availability. Seabird distributions get aggregated into a single cumulative sensitivity map with weightings based on sensitivity to offshore wind turbines. Each site (i.e. pixel on the map) can then be plotted in variable space as a tradeoff between wind profitability versus bird sensitivity. Each of these sites can be assigned a new utility value as a function of each axes, i.e. maximizing wind profitability while minimizing bird sensitivity. This new utility value can then be mapped out in space. Cetacean distributions are also aggregated into a cumulative sensitivity map, except weights are by extinction risk and a map is made for each month to capture variability in migratory patterns that could be differentially affected by episodic pile driving. These general processes (Fig 2) are given more in-depth treatment throughout the rest of the methods.

### Study area: Mid-Atlantic coast of the US

**Fig 3:**
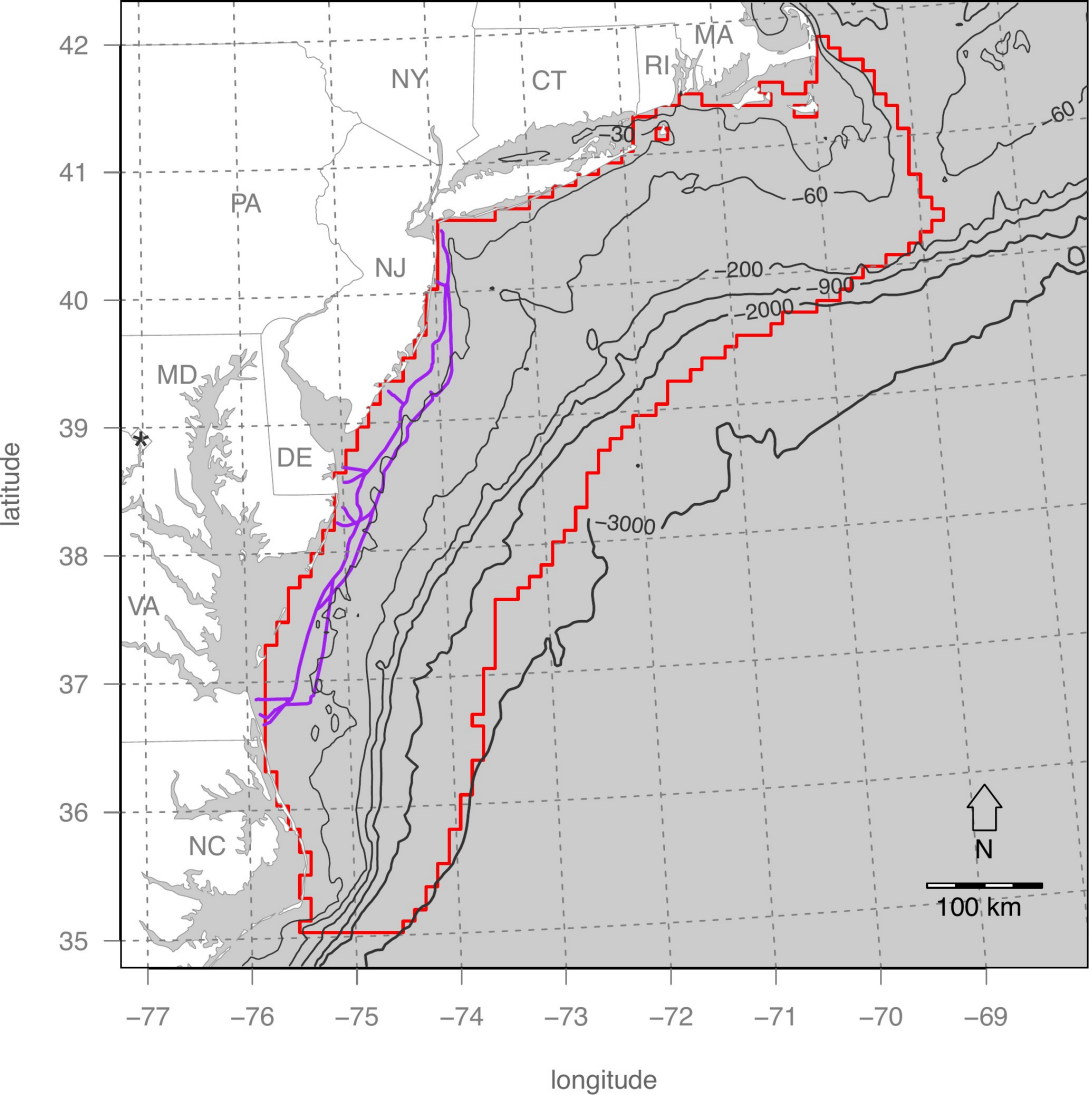
Mid-Atlantic offshore study area (red) and proposed Atlantic Wind Connection transmission leasing facility (blue). The study area is delimited by the availability of bird density data from the Atlantic Offshore Seabird Dataset Catalog. The pixelated edge is determined by the 10 km grid cells of the cetacean density surfaces (Roberts et al. 2016) in Albers Equal Area projection.

The Mid-Atlantic continental shelf of the US presents an opportune area for OWED given its strong offshore winds and proximity to densely populated coastal areas. The Atlantic Wind Connection, a Google backed offshore transmission grid in its early planning phase, could significantly lower costs to OWED leasees. Species densities are available for cetaceans [20] and birds (Atlantic Offshore Seabird Dataset Catalog; see Winship et al. [21] for latest). The study area is defined by the best available bird density surfaces at the time of the analyses (2015) (Fig 3).

### Wind energy valuation

The net present value (NPV) for each 10 km pixel site was estimated using the Offshore Wind Energy Production model from the InVEST Toolbox version 3.2 [22,23]. The candidate wind farm consists of the default InVEST configuration of 80 x 5MW turbines (400 MW capacity farm) with a hub height at 90m evaluated over a lifetime of 20 years. The NPV for a wind farm in the given pixel is determined by the gross revenues from wind energy (*R*_*t*_) minus the costs (C_*t*_) annualized (*t*) over the lifetime (*T*) of the wind farm modified by the discount rate (*i*) or weighted average cost of capital (1). A discount rate of 5% was applied per White et al. [6].

$$NPV=\sum_{t=1}^{T}(R_{t}-C_{t}){\left(1+i\right)}^{-t}$$

In terms of siting, revenue is largely determined by wind speed at hub height and costs by transmission distance to the grid. Since grid connection points are not made publicly available, distance to shoreline serves as a proxy. An additional 4 km to connect from shore to the grid was applied for all sites. An alternate scenario considering access to the Atlantic Wind Connection transmission reduced this distance to shore but did not consider additional (as yet unknown) leasing costs for its use.

Parameter coefficients (*β*_0,1_) to model transmission cost (*TransCost*) based on megawatt size of the wind farm (*MW*) and total cable laid (*TotCable*) were estimated by fitting costs available via literature search by the InVEST team (2).

$$TransCost=\beta_{0}MW+\beta_{1}TotCable$$

Separate coefficients were modeled based on an assumption of switching from alternating current (AC) to direct current (DC) at 60 km or greater distance per the InVEST Offshore Wind Energy Production documentation^1^.

Gross revenues (*R*) are derived from wind power by multiplying the price per kWh with the annual amount of kWh produced by the wind farm (*E*). This wind production is based on the individual turbine output (*O*) multiplied by the number of turbines (n = 80). Individual turbine output is based on the default InVEST parameters for the 5 MW turbine configuration (cut-in at 3 ms^-1^; rated windspeed at 12.5 ms^-1^; cut-out at 30 ms^-1^; rotor diameter of 116m) to describe a polynomial of power (5 MW) over a range of wind speed (cut-in to cut-out). Wind speed is estimated at hub height from the reference surface using a power curve based on a fitted Weibull distribution (see InVEST documentation at http://data.naturalcapitalproject.org/nightly-build/invest-users-guide/html/wind_energy.html). Wind speed at the ocean surface reference height is provided through InVEST from NOAA’s National Weather Service provides hindcast reanalysis (http://polar.ncep.noaa.gov/waves/index2.shtml) at half degree spatial resolution from 1999 to present.

Greater depths increase the cost of foundations and installation due to more required material but is not modeled here due to lack of published data for establishing an explicit relationship. A $2 M installation cost per turbine is applied to all sites equally. The jacketed foundations generally required for a 5 MW turbine are more expensive than the less robust monopole foundations used for 3.6 MW turbines. Floating structures open the possibility of going to still greater depths but are still in the demonstration phase.

Although the majority of costs are for installation, operations and maintenance account for a fraction of the capital expenditure annually. The default 3.5% value was applied.

### Bird distribution and sensitivity score

Density distributions for 27 individual bird species were downloaded from the Avian Average Annual Abundances [24,25] available at MarineCadastre.gov. These density maps were matched with species having sensitivity to OWED from a recent UK-based study on bird sensitivity to OWED [11] to yield 21 species for analysis (Table 1). The 6 species having density distributions and missing a sensitivity value from Bradbury et al. [11] were dropped from the analysis but are all of Least Concern for extinction risk according to the IUCN RedList: Bonaparte's gull (*Chroicocephalus philadelphia*), double-crested cormorant (*Phalacrocorax auritus*), laughing gull (*Larus atricilla*), red phalarope (*Phalaropus fulicarius*), ring-billed gull (*Larus delawarensis*), and surf scoter (*Melanitta perspicillata*).

**Table 1:** Bird sensitivity to OWED based on the maximum sensitivity of collision or displacement, per Bradbury et al. [11].

Common	Scientific	Sensitivity	Rank	Value
Great Black-backed Gull	*Larus marinus*	Collision	Very high	5
Herring Gull	*Larus argentatus*	Collision	Very high	5
Black Scoter	*Melanitta nigra*	Displacement	High	4
Black-legged Kittiwake	*Rissa tridactyla*	Collision	High	4
Common Loon	*Gavia immer*	Displacement	High	4
Northern Gannet	*Morus bassanus*	Collision	High	4
Red-throated Loon	*Gavia stellata*	Displacement	High	4
Common Eider	*Somateria mollissima*	Displacement	Moderate	3
Common Tern	*Sterna hirundo*	Collision	Moderate	3
Razorbill	*Alca torda*	Displacement	Moderate	3
Roseate Tern	*Sterna dougallii*	Both	Moderate	3
White-winged Scoter	*Melanitta fusca*	Displacement	Moderate	3
Leach's Storm Petrel	*Oceanodroma leucorhoa*	Collision	Low	2
Long-tailed Duck	*Clangula hyemalis*	Displacement	Low	2
Pomarine Jaeger	*Stercorarius pomarinus*	Collision	Low	2
Cory's Shearwater	*Calonectris diomedea*	Both	Very low	1
Dovekie	*Alle alle*	Both	Very low	1
Great Shearwater	*Puffinus gravis*	Both	Very low	1
Northern Fulmar	*Fulmarus glacialis*	Both	Very low	1
Sooty Shearwater	*Puffinus griseus*	Both	Very low	1
Wilson's Storm Petrel	*Oceanites oceanicus*	Both	Very low	1

Bird density maps are based on scientific surveys in the US Atlantic compiled [25] since 1978 into The Compendium of Avian Information in the US Atlantic Outer Continental Shelf [24] by NOAA/NOS/NCCOS working with the USGS Patuxent Wildlife Research Center for BOEM. Density (individuals per 2.5 nm strip width) were calculated seasonally and then averaged across the year.

Bird sensitivity to OWED developed by Bradbury et al. [11] separated collision risk from displacement impacts similar to Furness et al. [10].

collisionriskscore=a*(m+t+n)/3

Terms contributing to collision risk are flight altitude (*a*), flight maneuverability (*m*), percentage of time flying (*t*), and nocturnal flight activity (*n*).

displacementscore=[(d*h)*c]/10

Displacement score is determined by disturbance from wind farm structures, ship and helicopter traffic (*d*), habitat specialization (*h*) and conservation importance (*c*). Most terms are based on taxonomic expertise. The conservation importance score was based on UK-based measures: Birds Directive status, percent biogeographic population in English waters, adult survival rate and UK threat status.

The maximum of either collision risk or displacement score per species was used to arrive at the Atlantic study weights (Table 1), similar to Bradbury et al. (2014).

birdsensitivity=max(collisionriskscore,displacementscore)

Per 10 km pixel, the average bird density was log-transformed, multiplied by bird sensitivity, and averaged across all species (*S*).

$$bird\;pixel\;score=\sum_{bird}^{B}\log\left({density}_{bird}+1\right)*{sensitivity}_{bird}/B$$

### Cetacean distribution and conservation status

Cetacean distributions were gathered from a recently published study [20] describing density of cetaceans for 26 species and 3 guilds in the US Atlantic using survey data from boats and planes over a 23 year period with a variety of habitat predictors, including depth, temperature, wind, eddies, and productivity.

Impacts to cetaceans from OWED are not well described but understood to be mostly from intense acoustic energy during the construction phase from pile driving [16,26]. The species-specific responses of cetaceans are known for very few marine mammals, and have only been modeled in spatially explicitly detail for the harbor seal [27,28] and harbor porpoise [19,29] due to their common occurrence in European waters where the majority of OWED facilities have been installed. In the absence of a sensitivity index to OWED akin to the birds, conservation status was used as the sole measure of sensitivity to OWED. The NatureServe conservation status [30] was preferred because of greater specificity to the study area, versus many species listed as Data Deficient for the global IUCN RedList (Table 2). Scores were scaled 1 to 100 (Table 3) and applied to species, with averages taken for 3 guilds.

**Table 2:** Conservation status score by species using NatureServe. Species are listed amongst one of four large groups: baleen whales, beaked and sperm whales, large delphinoids and small delphinoids. For the 3 guilds (beaked whales, *Kogia* whales and pilot whales), species scores were averaged across member species. IUCN extinction risk categories were not used because many were data deficient (DD) (other codes: least concern (LC), vulnerable (VU) and endangered (EN)). The Endangared Species Act (ESA) was similarly limiting in providing a range of species weights based on conservation concern.

Common	Scientific	IUCN	ESA	NatureServe	Score
***Baleen whales***					
Blue whale	*Balaenoptera musculus*	VU	EN	G3G4	38
Bryde's whale	*Balaenoptera edeni*	DD		G4	26
Fin whale	*Balaenoptera physalus*	EN	EN	G3G4	38
Humpback whale	*Megaptera novaeangliae*	LC		G4	26
Minke whale	*Balaenoptera acutorostrata*	LC		G5	1
North Atlantic right whale	*Eubalaena glacialis*	EN	EN	G1	100
Sei whale	*Balaenoptera borealis*	EN	EN	G3	51
***Beaked and sperm whales***					
Beaked whales					41
Blainville's beaked whale	*Mesoplodon densirostris*	DD		G4	26
Cuvier's beaked whale	*Ziphius cavirostris*	LC		G4	26
Gervais beaked whale	*Mesoplodon europaeus*	DD		G3	51
Sowerby's beaked whale	*Mesoplodon bidens*	DD		G3	51
True's beaked whale	*Mesoplodon mirus*	DD		G3	51
*Kogia* whales					26
Dwarf sperm whale	*Kogia sima*	DD		G4	26
Pygmy sperm whale	*Kogia breviceps*	DD		G4	26
Northern bottlenose whale	*Hyperoodon ampullatus*	DD		G4	26
Sperm whale	*Physeter macrocephalus*	VU	EN	G3G4	38
***Large delphinoids***					
False killer whale	*Pseudorca crassidens*	DD		G4	26
Killer whale	*Orcinus orca*	DD	EN	G4G5	14
Melon headed whale	*Peponocephala electra*	LC		G4	26
Pilot whales					1
Pilot whale, long-finned	*Globicephala melas*	DD		G5	1
Pilot whale, short-finned	*Globicephala macrorhynchus*	DD		G5	1
***Small delphinoids***					
Risso's dolphin	*Grampus griseus*	LC		G5	1
Atlantic spotted dolphin	*Stenella frontalis*	DD		G5	1
Atlantic white-sided dolphin	*Lagenorhynchus acutus*	LC		G4	26
Common bottlenose dolphin	*Tursiops truncatus*	LC		G5	1
Clymene dolphin	*Stenella clymene*	DD		G4	26
Fraser's dolphin	*Lagenodelphis hosei*	LC		G4	26
Harbor porpoise	*Phocoena phocoena*	LC		G4G5	13
Pantropical spotted dolphin	*Stenella attenuata*	LC		G5	1
Rough-toothed dolphin	*Steno bredanensis*	LC		G4	26
Short-beaked common dolphin	*Delphinus delphis*	LC		G5	1
Spinner dolphin	*Stenella longirostris*	DD		G5	1
Striped dolphin	*Stenella coeruleoalba*	LC		G5	1
White-beaked dolphin	*Lagenorhynchus albirostris*	LC		G4	26

**Table 3:** Lookup values to assign conservation score based on NatureServe conservation status.

NatureServe	Description	Score
G1	Critically imperiled	100
G1G2		87
G2	Imperiled	75
G2G3		63
G3	Vulnerable	51
G3G4		38
G4	Apparently secure	26
G4G5		13
G5	Secure	1

Unlike the bird distribution data, the cetacean predictions are available at monthly time steps. Cells containing the lowest 1% of total density were masked from analysis. Individual species densities (*d*) per 10 km^2^ pixel (*i*) were rescaled as the difference from mean value ($\bar{d}$) over the standard deviation of the density within the study area. Species scores were averaged across all species (*S*) to arrive at a cetacean score per pixel and month.

cetaceanscorei=∑sSds,i−d¯sd(d)*csS

The cetacean score was finally rescaled 0 to 1, minimum to maximum.

### Evaluating tradeoffs as a utility function

Deciding to site offshore wind energy development is based on weighing tradeoffs between wind energy profitability and species conservation. Each site can be examined according to a tradeoff plot with either value on the axis. Deciding how much influence species conservation will be imposed at the loss of wind profitability is a societal decision involving industry, government regulatory agencies and other stakeholders. Ideally, solutions exist which favor both goals, the preferred “win-win” scenario. We can quantitatively evaluate this tradeoff over a range of utility functions (Equation 8).

u=a*WindProfitability−(1−a)*BirdSensitivity

The weighting term (*a*) then indicates a preference of wind profitability versus bird sensitivity. Eventually this term could be implemented as sliders in a user interface. For the purposes of these initial results, we simulated over a naïve range of the weighting term (*a*) from 0 to 1 at a step of 0.1. The utility (*u*) was then averaged to arrive at an average utility ($\bar{u}$) per site.

So then what is a reasonable range of each axis and overall utility to suggest for OWED? Garthe & Hüppop [9] designated the top 60% of bird sensitivity scores as areas of “concern” and top 20% as areas of “major concern”. We adopted these quantiles across each axes and overall utility as a visual guide.

Since the values of each axes (wind profitability and bird sensitivity) were normalized (0 to 1), it is worth pointing out that the relationship between these terms is dependent on the extent of the study area and the values contained therein.

### Spatiotemporal decision support system

All the analysis, besides the wind energy valuation with InVEST, was coded in the free, open-source, cross-platform statistical programming language R [31]. The spatial-temporal decision support system web-based interface was developed with the R package Shiny [32] using leaflet [33] for interactive mapping and ggvis [34] with plotly [35] for interactive plotting. The code is freely available (https://github.com/bbest/siting).

## Results

### Map of wind energy valuation

**Fig 4:**
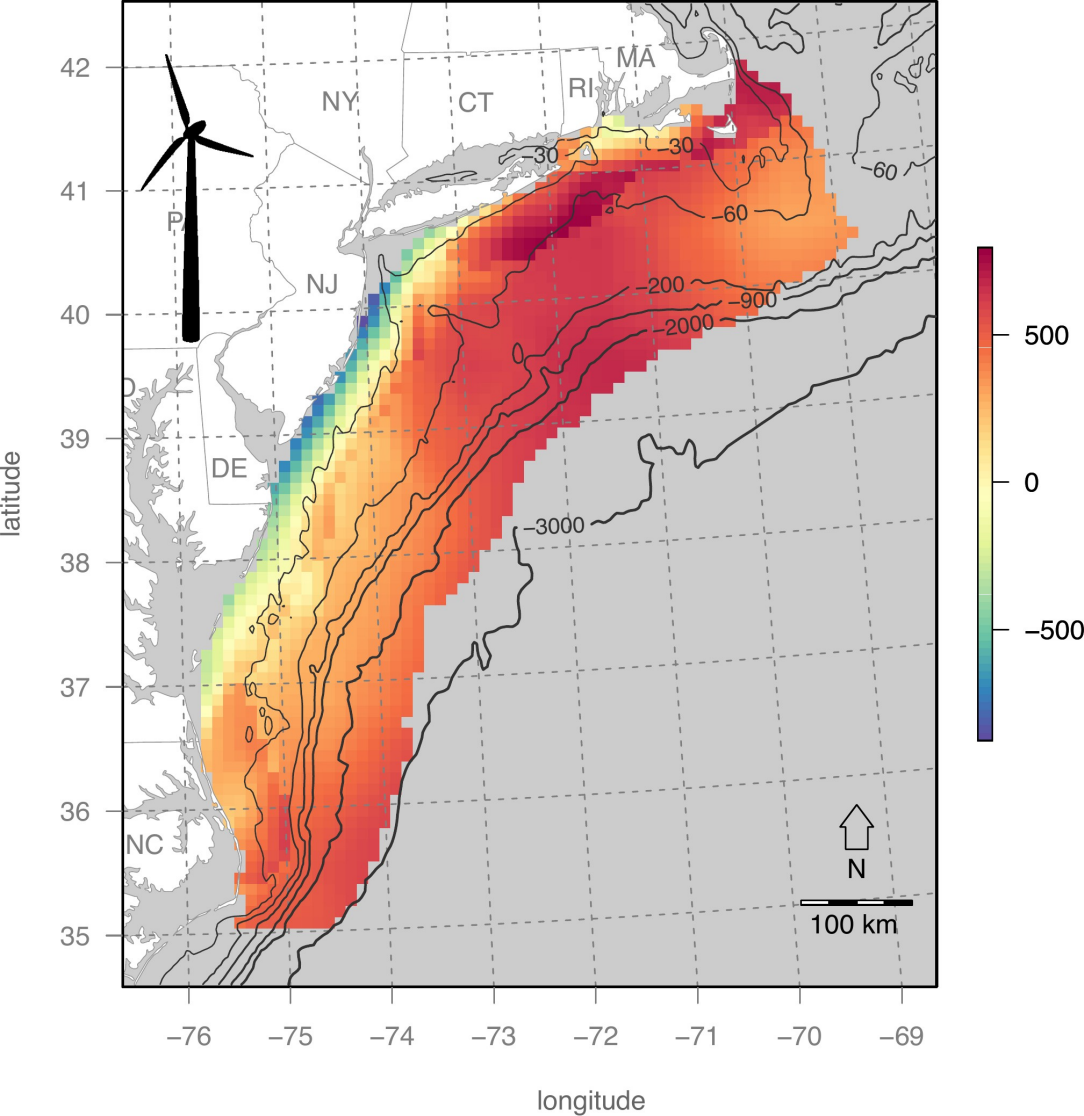
Wind energy valuation (net present value in $US millions). Bathymetric depths are contoured in light gray.

**Fig 5:**
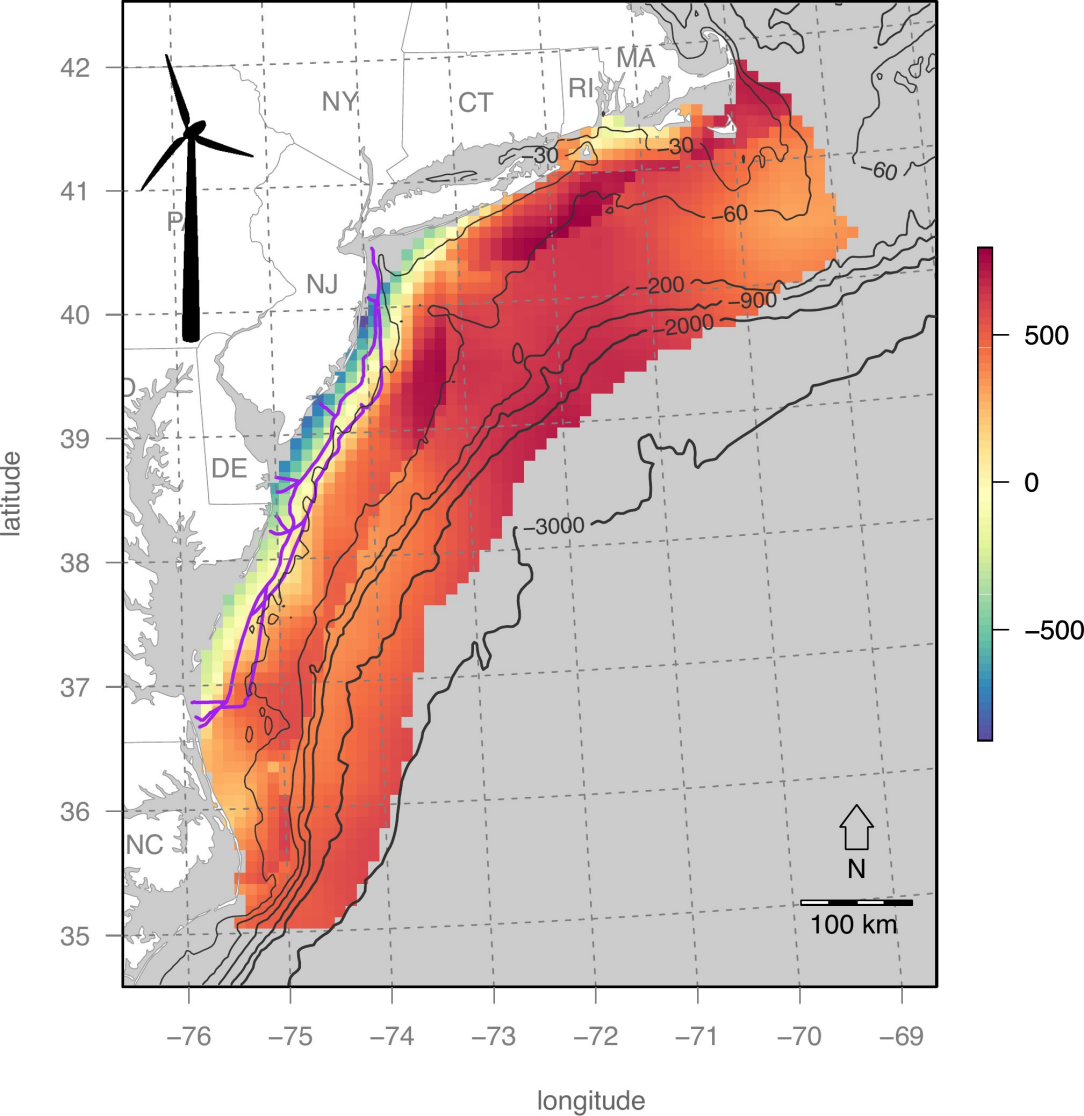
Wind energy valuation (net present value in $US millions) with access to the Atlantic Wind Connection (purple lines).

The net present value of OWED for the US Mid-Atlantic (Fig 4) shows a trend of increasing value offshore and more northern latitudes that is a driven by higher wind speeds in these areas. Most coastal pixels near New Jersey and Delaware are even negative, thus unrealistic for investment. This pattern is consistent when modeling with the Atlantic Wind Connection (Fig 5) with higher profit to be gained near the transmission line and further offshore.

### Map of bird sensitivity score

**Fig 6:**
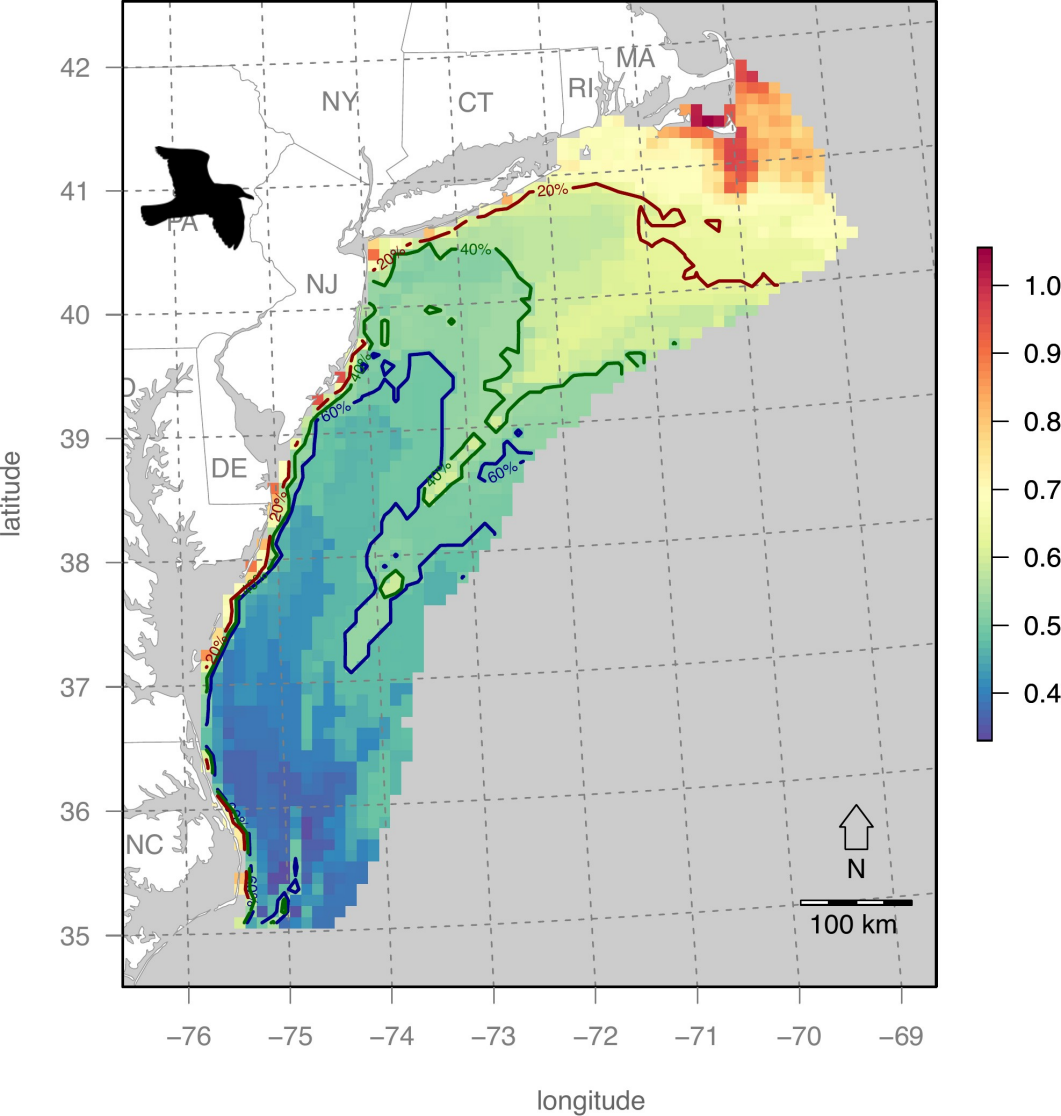
Cumulative bird sensitivity to offshore wind energy development. Bird sensitivity dramatically increases with latitutude and slightly further offshore. Contours of the the top 20% and 60% quantile areas denote areas dubbed as “major concern” and “concern” respectively, leaving only the remaining bluest areas offshore from North Carolina (NC) as the only “least concern” areas, per the classification scheme of Garthe & Hüppop (2004).

Bird sensitivity exhibits a strong latitudinal gradient with Massachusetts to the north having the highest values and lowest offshore from North Carolina (Fig 6).

This average seabird pattern is determined by the fact that the majority of individual bird distributions (S1–S21 Figs) dominate the northern region, particularly the ones with the highest OWED sensitivity value of 5: Great Black-backed Gull (*Larus marinus*; S1 Fig); Herring Gull (*Larus argentatus*; S2 Fig). There are, however exceptions to this pattern, such as the coastal, southerly presence of the Black Scoter (*Melanitta nigra*; S3 Fig), Red-throated Loon (*Gavia stellate*; S7 Fig), offshore presence of Leach's Storm Petrel (*Oceanodroma leucorhoa*; S13 Fig) and bimodal presence both North and South of Cory’s Shearwater (*Calonectris diomedea*; S16 Fig).

### Map of cetacean conservation status

**Fig 7:**
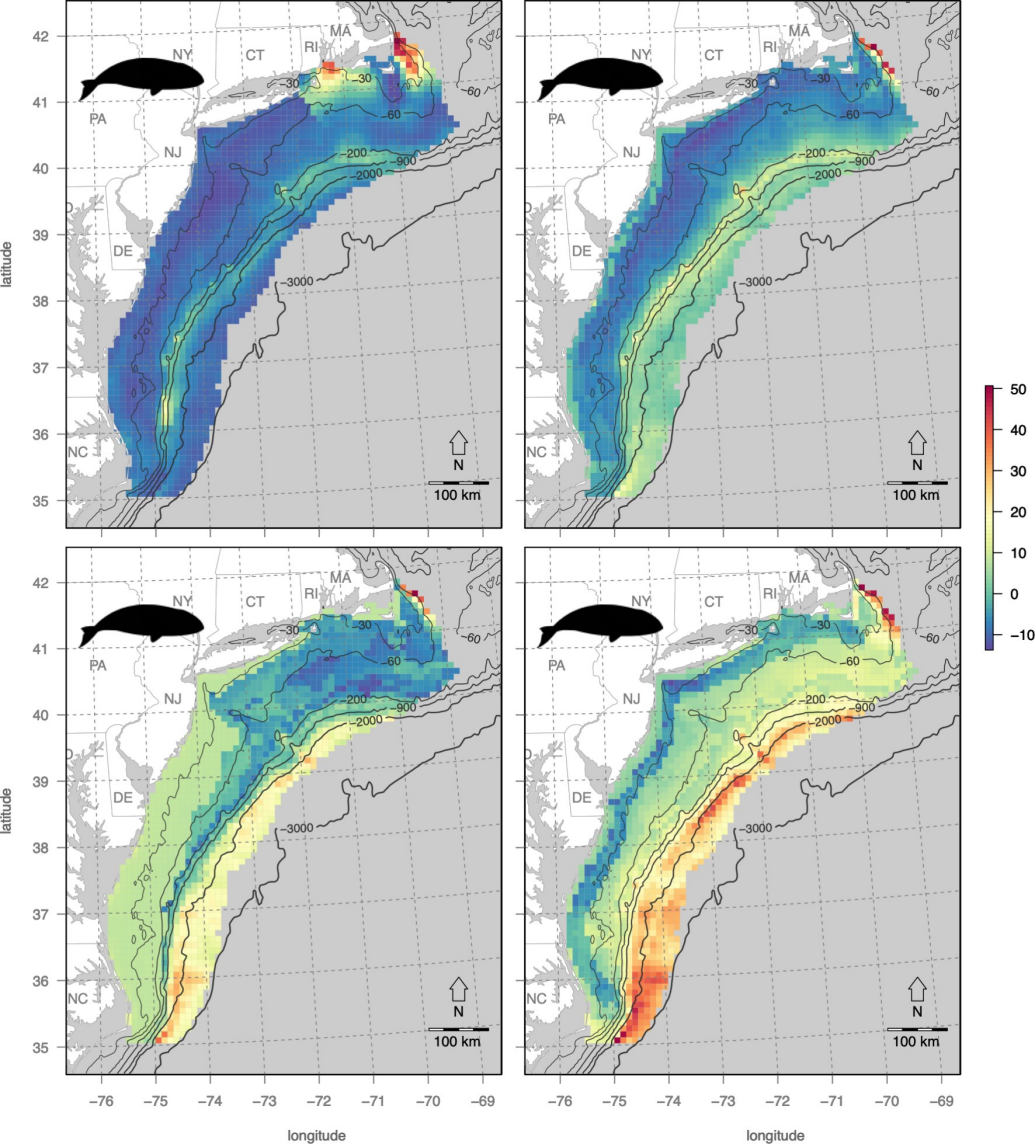
Cetacean sensitivity for specific months with site labels.

Migration patterns of cetaceans in the US Mid-Atlantic exhibit considerable variation between months (Fig 7). March is most intensely concentrated near the Gulf of Maine, May on the northern fringe, August diffuse throughout, and November peaked on either end of the study area with a consistent offshore signature. These patterns are largely driven by the North Atlantic right whale (*Eubalaena glacialis)*, which is critically imperiled with a total population last estimated at 529 individuals [36] and migrates south in the winter to calving grounds in Florida and north to forage in the summer in the Gulf of Maine, with some populations maintaining residency in Gulf of Maine year-round [20]. The overall cetacean sensitivity map, however, represents an average of all species included in the analyses (Table 2), so should not be confused with deeming any given area safe from potential presence of any endangered species, including the North Atlantic right whale.

### Tradeoffs in space with birds

**Fig 8:**
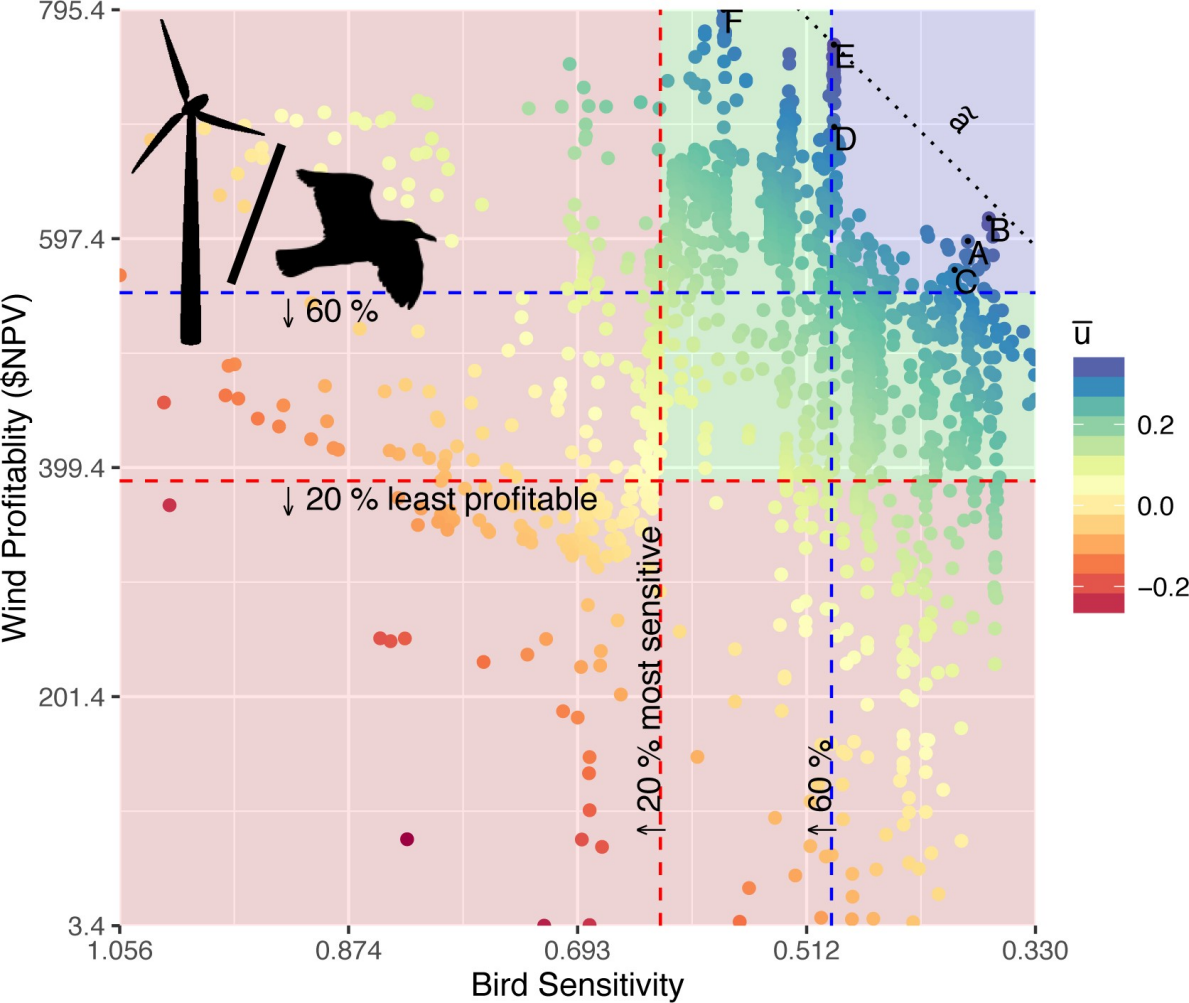
Tradeoff for all sites between bird sensitivity and wind profitability as net present value (NPV) in $US millions. Sites with negative NPV were excluded from this plot. Values were rescaled before calculating average utility ($\bar{u}$) of each site from 11 simulations of the utility function ranging *a* in Equation 8 from 0 to 1. The slope of the median $\tilde{a}$ is shown as a dotted line passing through the highest utility site E. The red quadrant corresponds to the sites with the least 20% of profitability or the most 20% of bird sensitivity. The upper right blue quadrant corresponds with sites excluding the 60% least profitable and 60% most sensitive, hence a preferred subset for development of offshore wind energy.

By plotting individual pixel values by management objectives, which are maximizing OWED profitability versus minimizing bird sensitivity, the tradeoff plot highlights sites that most match each objective (Fig 8). The upper right quadrant is most desirable for selecting the most profitable, least sensitive sites. This is further quantified by creating quadrants from quantile values along each axis, per the 20% and 60% quantiles introduced by Garthe & Hüppop [9].

**Fig 9:**
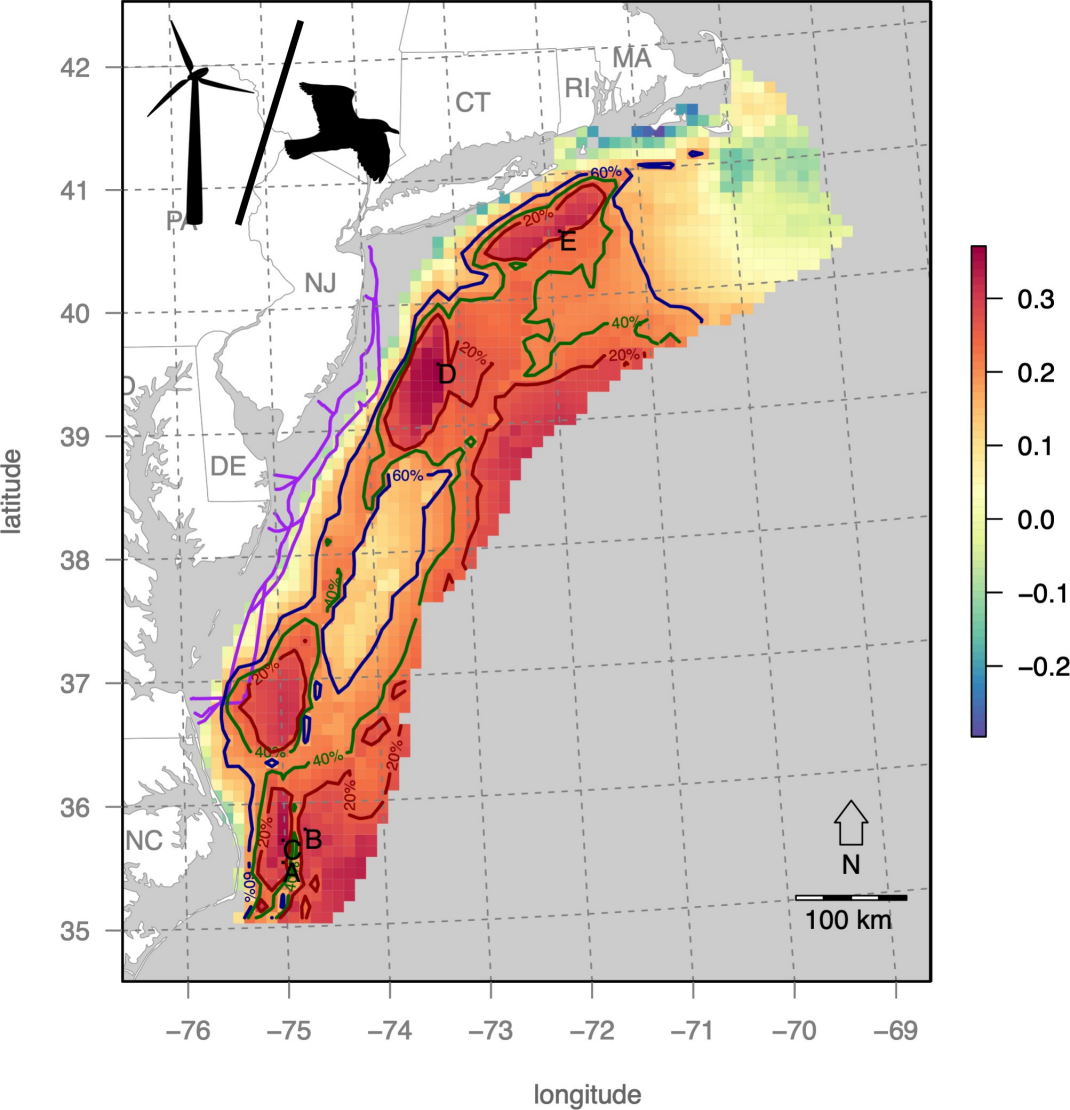
Map of average utility from simulation. Contours for the top 20%, 40% and 60% quantiles of average utility. Site labels A-E are of highest utility within the 20% contour area and correspond with labels in the tradeoff plot (Fig 8) and table of values (Table 4).

With the average utility value combining both objectives, these values are then plotted back onto the map to highlight areas of greatest prospective interest (Fig 9). Contouring the top 20% of sites reveals 6 hotspots for OWED that maximize wind profitability and minimize bird sensitivity. By labeling the highest value pixels within each of these hotspot areas, the pixel values can be compared with others and ranked (Table 4).

**Table 4:** Values of bird sensitivity, wind profitability (net present value in $US millions), average utility across simulations and sorted by overall rank for selected sites, corresponding to labels in the tradeoff plot (Fig 8) and average utility map (Fig 9).

Label	Lon	Lat	Bird Sensitivity	Wind Profitability	Average Utility	Rank
E	-73.24	39.51	0.49	765.2	0.371	1
B	-75.02	35.70	0.37	615.1	0.361	4
A	-74.25	35.41	0.38	595.4	0.337	24
F	-71.88	40.53	0.58	795.4	0.330	28
D	-72.36	38.75	0.49	694.0	0.326	36
C	-74.98	36.78	0.39	570.4	0.314	62

**Fig 10:**
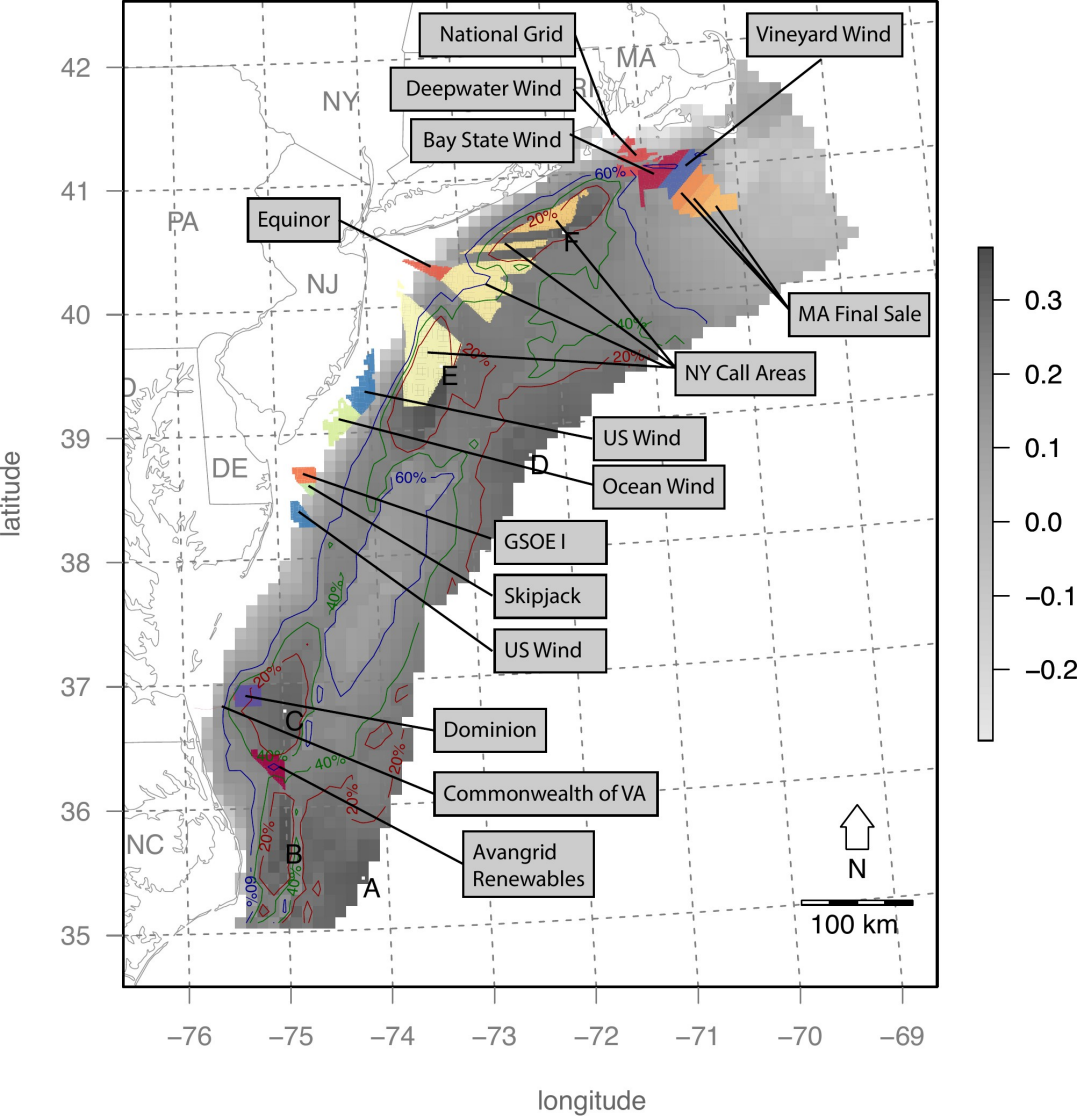
Map of BOEM Wind Energy Areas and Leases (as of October, 2018) in the context overall utility in grayscale with contours and site labels similar to Fig 9.

These tradeoffs can then be compared to existing BOEM leases and call areas (Fig 10). Some of these hotspots overlap existing call areas, such as the southern and two northernmost New York Bight Call Areas where BOEM is engaged in active planning (https://www.boem.gov/New-York). In contrast, the Massachusetts Final Sale Notice areas have low overall utility, driven by high interaction with birds (Fig 6) despite high wind profitability (Fig 5).

### Tradeoffs in time with cetaceans

**Fig 11:**
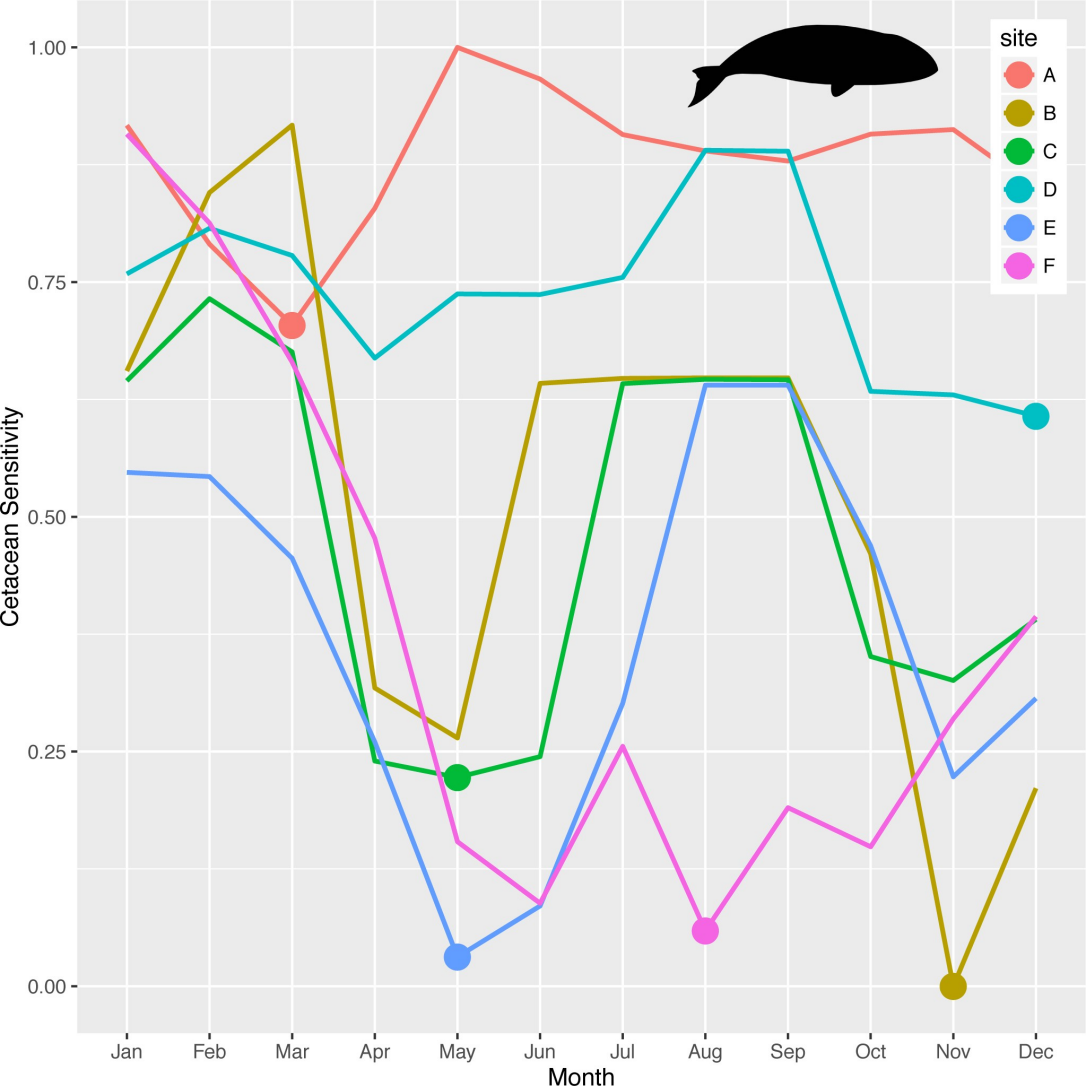
Cetacean sensitivity for specific months for identified sites (Table 4, Fig 9). The month with the minimum sensitivity is emphasized (filled circle marker) per site.

Once sites are selected in space based on maximizing long-term operational profitability of OWED and minimizing impacts on sensitive bird species, impacts to cetaceans can be evaluated over time. Sites selected previously based on highest overall utility (Fig 9) are next examined over time to identify months with the least impact on cetaceans to conduct pre-operational activities such as pile driving and seismic surveying (Fig 11). Sites A and D offshore, near the continental shelf break and Gulf Stream have relatively high, consistent cetacean risk throughout the year, however minimal months do occur in March for site A offshore from Cape Hatteras, NC and site D offshore from NJ. In contrast, sites B, C and E are at similar, nearer distances from shore and display a “W” pattern with the least sensitive month being November, May and May, respectively.

### Spatiotemporal decision support system

**Fig 12:**
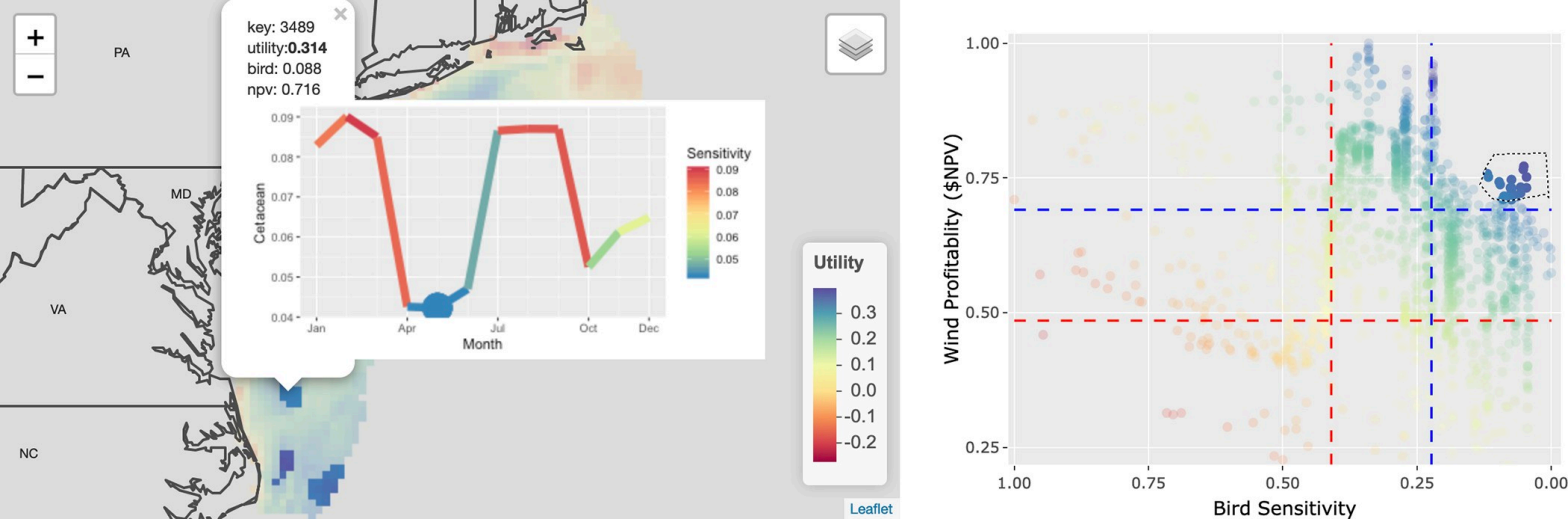
Spatiotemporal decision support interface showing interactive map on the left and tradeoff plot of bird sensitivity versus industry profitability on the right. The pixels of the map are colored by the utility function that maximizes profitability to industry while minimizing bird sensitivity. Clicking on a given pixel in the map will popup cetacean sensitivity over the year and highlight the month with the minimum sensitivity for timing harmful activities such as pile driving and seismic airgun surveying. Selecting rows in the table will highlight them on the tradeoff plot. Selecting points on the tradeoff plot will highlight them in the map. This application is available online at http://shiny.env.duke.edu/bbest/siting.

The spatiotemporal decision support system (SDSS; Fig 12) is highly interactive: pan and zoom in the map, lasso sites in the tradeoff plot to highlight the site pixels on the map, click on a site pixel in the map and get the cetacean sensitivity over time. Many other criteria, such as human use by military, fisheries and shipping industries beyond those modeled for this exercise are inevitably part of the planning process. This SDSS is therefore not comprehensive but enables exploration of alternative sites to deeply evaluate conservation and OWED industry concerns.

## Discussion

The recommended framework for prospecting offshore wind energy development is to consider areas that maximize profitability to offshore wind energy while minimizing to sensitivity to bird, since birds are exposed over the long-term operational phase of wind farms. Mapping the utility, which is described by the tradeoff plot, highlights sites that most efficiently meet both objectives. Subsequent planning for construction activities should be timed so as to minimize acoustic exposure to cetaceans of conservation concern. This too can be systematically quantified with a cetacean sensitivity plot per site over time (Figs 1 & 2).

Within the Mid-Atlantic study area, several areas were identified (Table 4; Fig 9) to have this optimal combination of high wind energy profitability and minimum seabird sensitivity. The New York Call Areas (sites E & F in Table 4 & Fig 10), where BOEM is currently engaging stakeholders (https://www.boem.gov/New-York), are particularly well optimized for minimal impact on seabirds with maximal profits to OWED. In contrast, the Massachusetts areas (Fig 10) are more prone to seabird sensitivity (Fig 6), so derive lower utility (Fig 9) despite higher OWED profitability (Fig 4). Other areas identified in the analysis as having optimal utility occur offshore North Carolina (sites A, B, C in Table 4 & Fig 10) and well offshore of New Jersey (site D in Table 4 & Fig 10). This offshore New Jersey site, however, is likely to be more costly given extra cost of materials at greater depth (versus equal fixed installation cost applied to the InVEST model) and costs due to transmission loss and/or repeater costs at further distances from shore (not captured in the InVEST model).

For the sites within optimal OWED/seabird utility areas (Table 4; Fig 9), the cetacean sensitivity analyses (Fig 11) determined the optimal month to conduct pile driving or other acoustical activities: March (site A), May (sites C, E), August (site F), November (site B) and December (site D). In a public letter (February 5, 2013) from the Natural Resource Defense Council (NRDC) to Deepwater Wind Block Island, LLC (DWW), NRDC requested that DWW alter their original pile driving schedule from April to another month that minimized potential interaction with North Atlantic right whales migrating between feeding grounds (Gulf of Maine) and calving grounds (southeastern US). This tool aims to systematically suggest environmentally responsible times to conduct these activities, and it could be further modified to capture species of concern individually.

This approach embodies the characteristics of sound ecosystem-based management: accounting for conservation of multiple species, while promoting sustainable marine industries, all within a user interface to solicit stakeholder feedback [37–39]. This approach is intended to inform decision makers and stakeholders and to provide substantial spatial and temporal decision-making support, which has been found lacking in environmental impact assessments for OWED [40]. The sites are ranked by highest average utility, which represent the best sites that meet both objectives across a set of utility functions that range from maximizing only conservation to only OWED profitability. For applying to other study areas, it is worth noting that the normalizing of species distributions and axes in the tradeoff plots renders results relative to absolute values within the given study area, so high utility values for one study area could be low and/or not comparable to another area (and vice versa).

Rather than necessarily dictating sites to develop offshore wind energy, outputs from this analysis enable BOEM and other agencies and related stakeholders to prioritize specific Mid-Atlantic lease blocks to minimize subsequent conservation obstacles involved in the environmental planning process. The most appropriate time for the implementation of this type of decision support tool is in the early stages of regional, multi-sector ocean planning (for example, see: Northeast Ocean Plan https://neoceanplanning.org/plan/; Mid-Atlantic Ocean Action Plan https://www.boem.gov/MidA-New/). During the more informal, pre-application phase of OWED siting, adjustments to the location and timing of development may be more flexible and amenable for consideration of decision support outcomes. Once a pool of lease areas has been selected, the range of allowable areas to be considered in the SDSS system will be more limited. However, the SDSS system can still be directly useful in the development of Environmental Impact Studies (EIS) and implementation plans [1] within regional lease area assessments.

The complex mass of input data (offshore wind, distance to grid connections, species densities, species migratory patterns, species conservation status, OWED sensitivities) are distilled into a holistic view where the optimal choices are clearly presented in both variable (i.e. tradeoff plot) and spatial (i.e. map of average utility per site) views. This effectively “games” stakeholders towards win-win solutions that serve to benefit both industry and environment. Interactivity in the SDSS (Fig 12) reveals the totality of the process, avoiding other “black box” approaches. The transparency of this system is expected to elicit stakeholder buy-in, which is critical for effective marine spatial planning [41–45].

As further research elucidates sensitivities of species to OWED, this framework can be expanded to accommodate new information. Ideally, future tools will assess not just relative concern but also estimate numbers of animals affected by proposed OWED based on the latest parameters related to species population dynamics and sensitivity to OWED [21,46,47]. Decision support tools of this type need to be regularly updated to reflect the most recent and accepted marine animal and marine environment information [48] and linked to the publicly available ocean planning data portals (e.g. Mid-Atlantic https://portal.midatlanticocean.org; Northeast https://northeastoceandata.org; and US https://marinecadastre.gov). In the future, it is hoped that population level impacts, i.e. potential biological removal estimates, will be incorporated for the most direct applicability to policy decisions [49,50].

## Supporting information

S1 Fig Seabird sensitivity map of Great Black-backed Gull (*Larus OWED*; marinus sensitivity value: 5). (TIFF)

S2 Fig Seabird sensitivity map of Herring Gull (*Larus argentatus*; OWED sensitivity value: 5). (TIFF)

S3 Fig Seabird sensitivity map of Black Scoter (*Melanitta nigra*; OWED sensitivity value: 4). (TIFF)

S4 Fig Seabird sensitivity map of Black-legged Kittiwake (*Rissa tridactyla*; OWED sensitivity value: 4). (TIFF)

S5 Fig Seabird sensitivity map of Common Loon (*Gavia immer*; OWED sensitivity value: 4). (TIFF)

S6 Fig Seabird sensitivity map of Northern Gannet (*Morus bassanus*; OWED sensitivity value: 4). (TIFF)

S7 Fig Seabird sensitivity map of Red-throated Loon (*Gavia stellate*; OWED sensitivity value: 4). (TIFF)

S8 Fig Seabird sensitivity map of Common Eider (*Somateria mollissima*; OWED sensitivity value: 3). (TIFF)

S9 Fig Seabird sensitivity map of Common Tern (*Sterna hirundo*; OWED sensitivity value: 3). (TIFF)

S10 Fig Seabird sensitivity map of Razorbill (*Alca torda*; OWED sensitivity value: 3). (TIFF)

S11 Fig Seabird sensitivity map of Roseate Tern (*Sterna dougallii*; OWED sensitivity value: 3). (TIFF)

S12 Fig Seabird sensitivity map of White-winged Scoter (*Melanitta fusca*; OWED sensitivity value: 3). (TIFF)

S13 Fig Seabird sensitivity map of Leach's Storm Petrel (*Oceanodroma leucorhoa*; OWED sensitivity value: 2). (TIFF)

S14 Fig Seabird sensitivity map of Long-tailed Duck (*Clangula hyemalis*; OWED sensitivity value: 2). (TIFF)

S15 Fig Seabird sensitivity map of Pomarine Jaeger (*Stercorarius pomarinus*; OWED sensitivity value: 2). (TIFF)

S16 Fig Seabird sensitivity map of Cory's Shearwater (*Calonectris diomedea*; OWED sensitivity value: 1). (TIFF)

S17 Fig Seabird sensitivity map of Dovekie (*Alle alle*; OWED sensitivity value: 1). (TIFF)

S18 Fig Seabird sensitivity map of Great Shearwater (*Puffinus gravis*; OWED sensitivity value: 1). (TIFF)

S19 Fig Seabird sensitivity map of Northern Fulmar (*Fulmarus glacialis*; OWED sensitivity value: 1). (TIFF)

S20 Fig Seabird sensitivity map of Sooty Shearwater (*Puffinus griseus*; OWED sensitivity value: 1). (TIFF)

S21 Fig Seabird sensitivity map of Wilson's Storm Petrel (*Oceanites oceanicus*; OWED sensitivity value: 1). (TIFF)
