# Supplementary figures and images for: Minimizing wildlife impacts for offshore wind energy development: Winning tradeoffs for seabirds in space and cetaceans in time
Source: PLoS One. 2019 May 14;14(5):e0215722. doi: 10.1371/journal.pone.0215722 (PMC6516727; doi:10.1371/journal.pone.0215722)

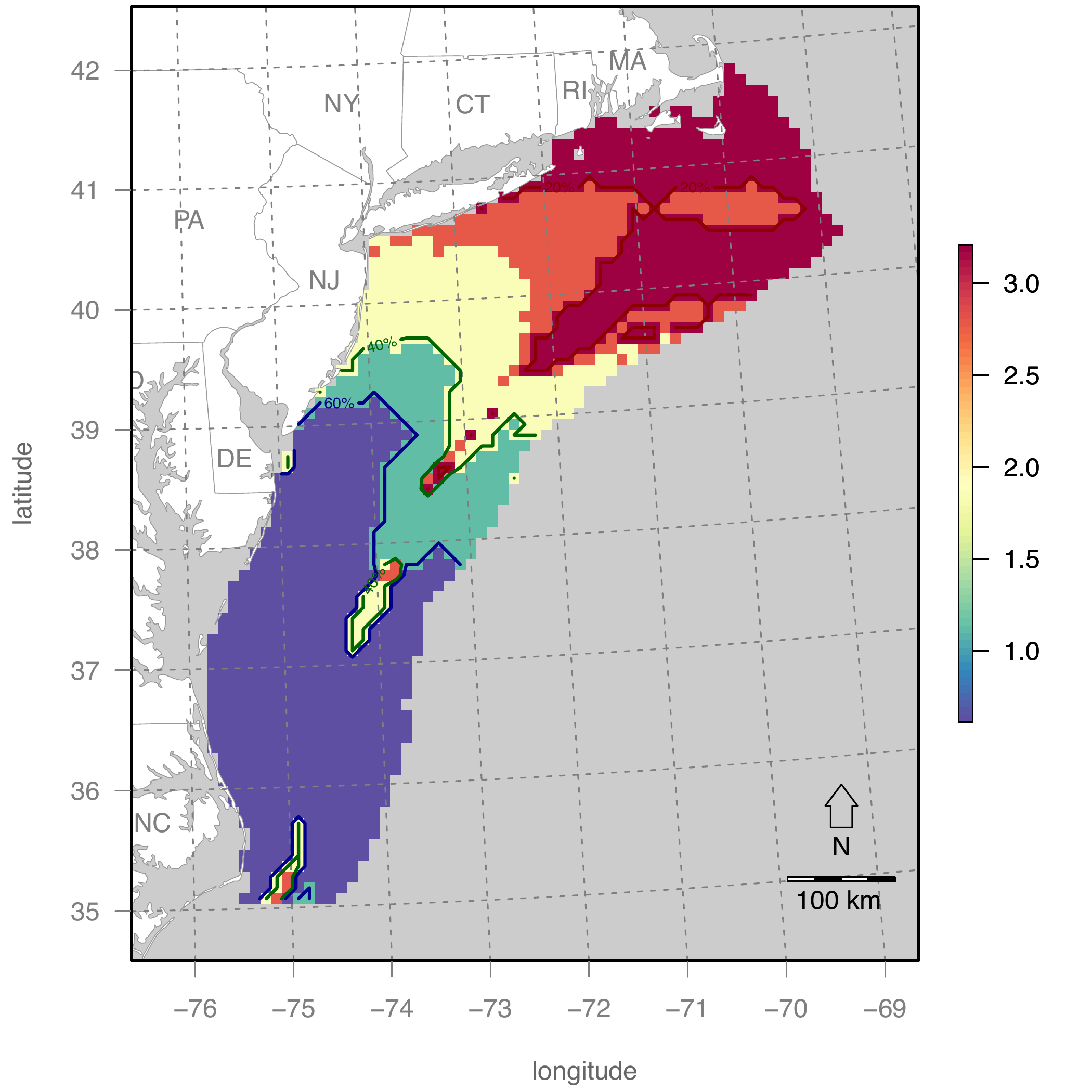

Supplement: S1 Fig — (TIFF) [file pone.0215722.s001.tiff]

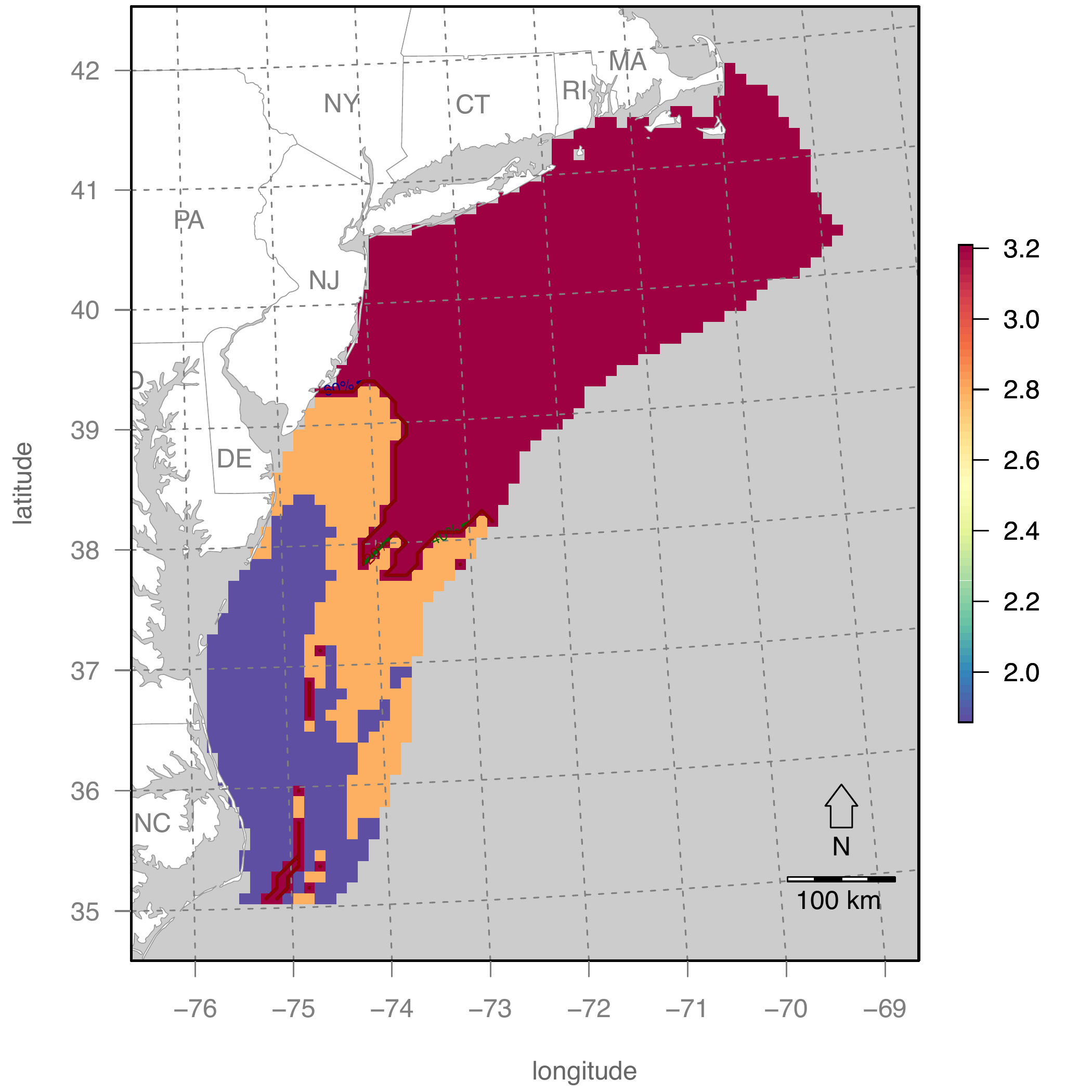

Supplement: S2 Fig — (TIFF) [file pone.0215722.s002.tiff]

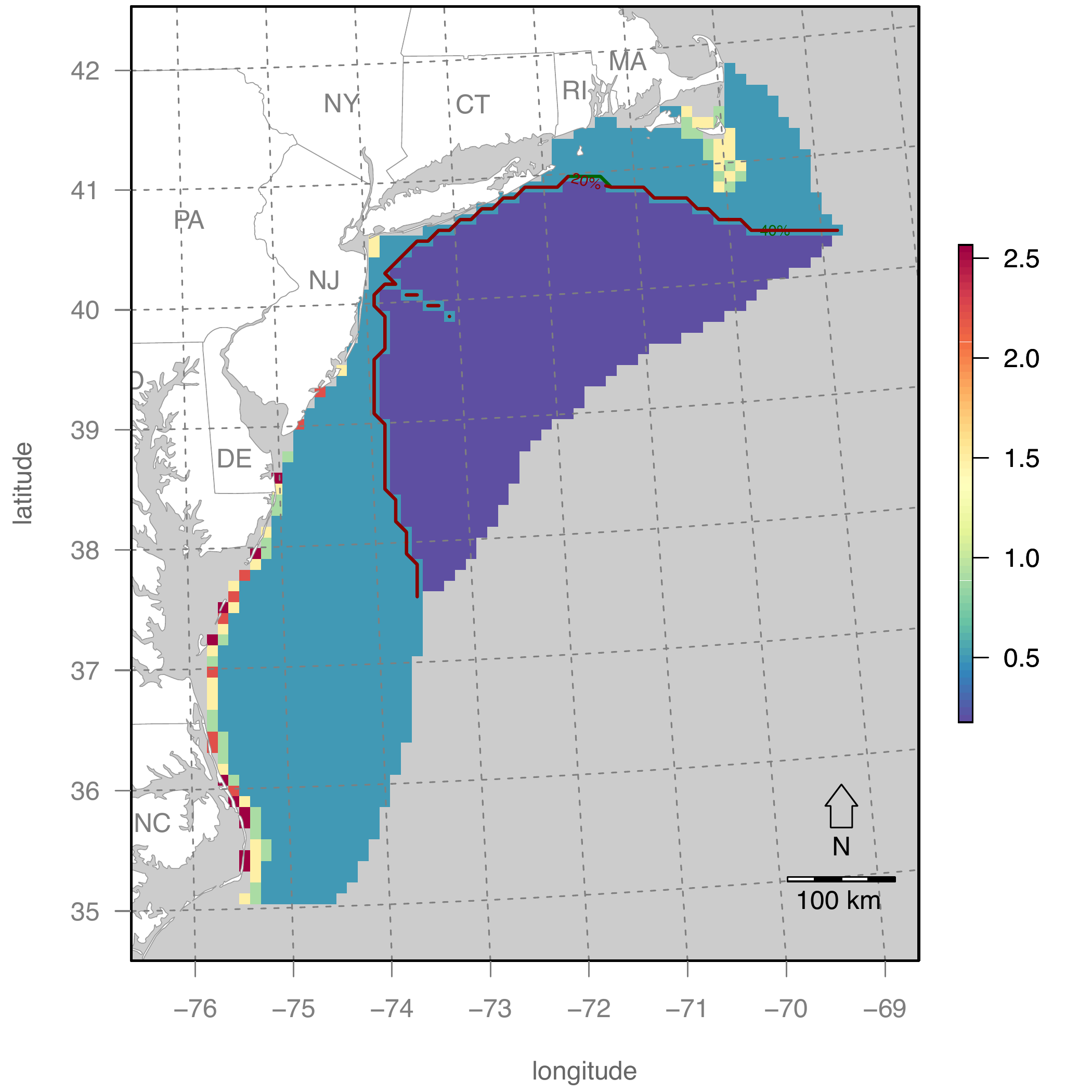

Supplement: S3 Fig — (TIFF) [file pone.0215722.s003.tiff]

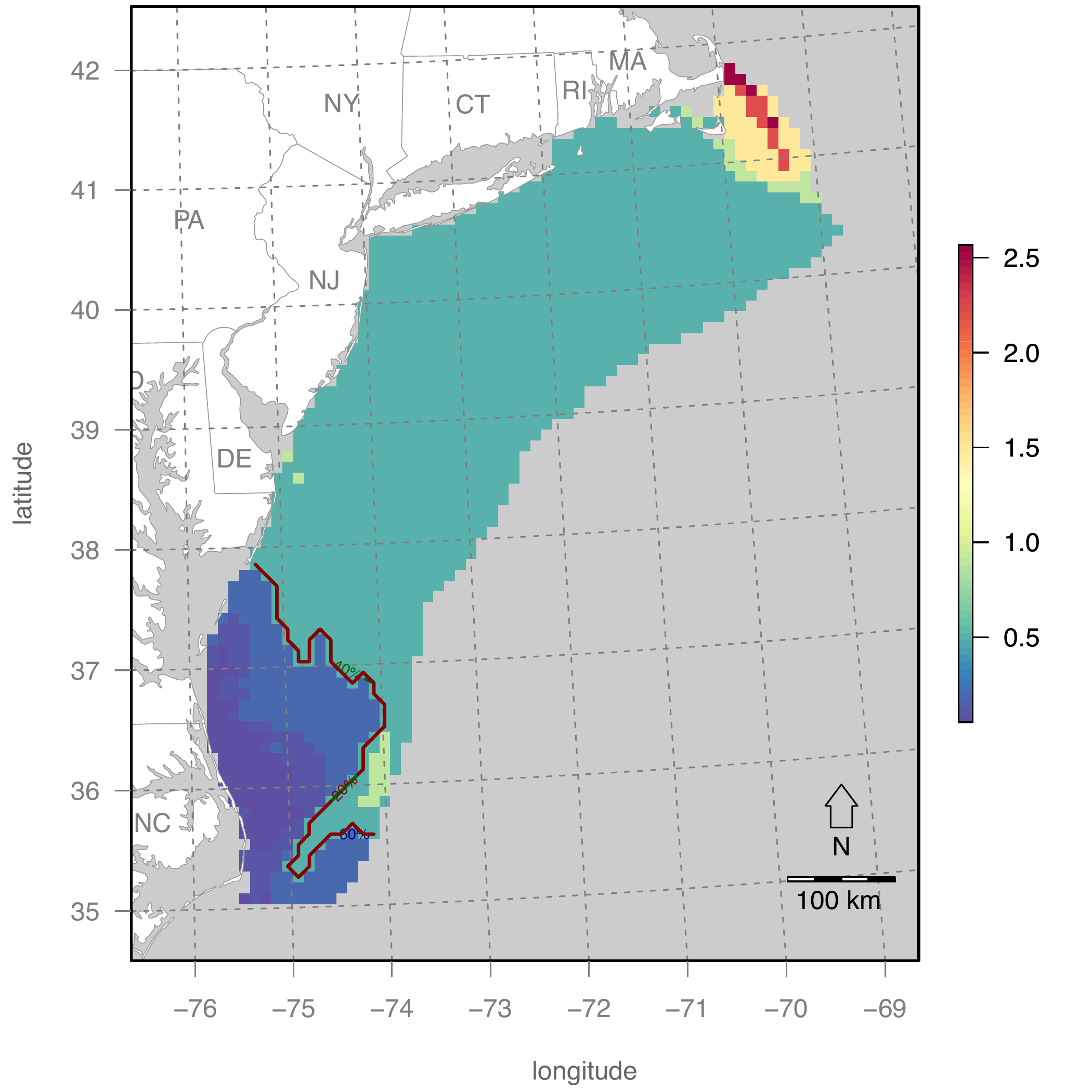

Supplement: S4 Fig — (TIFF) [file pone.0215722.s004.tiff]

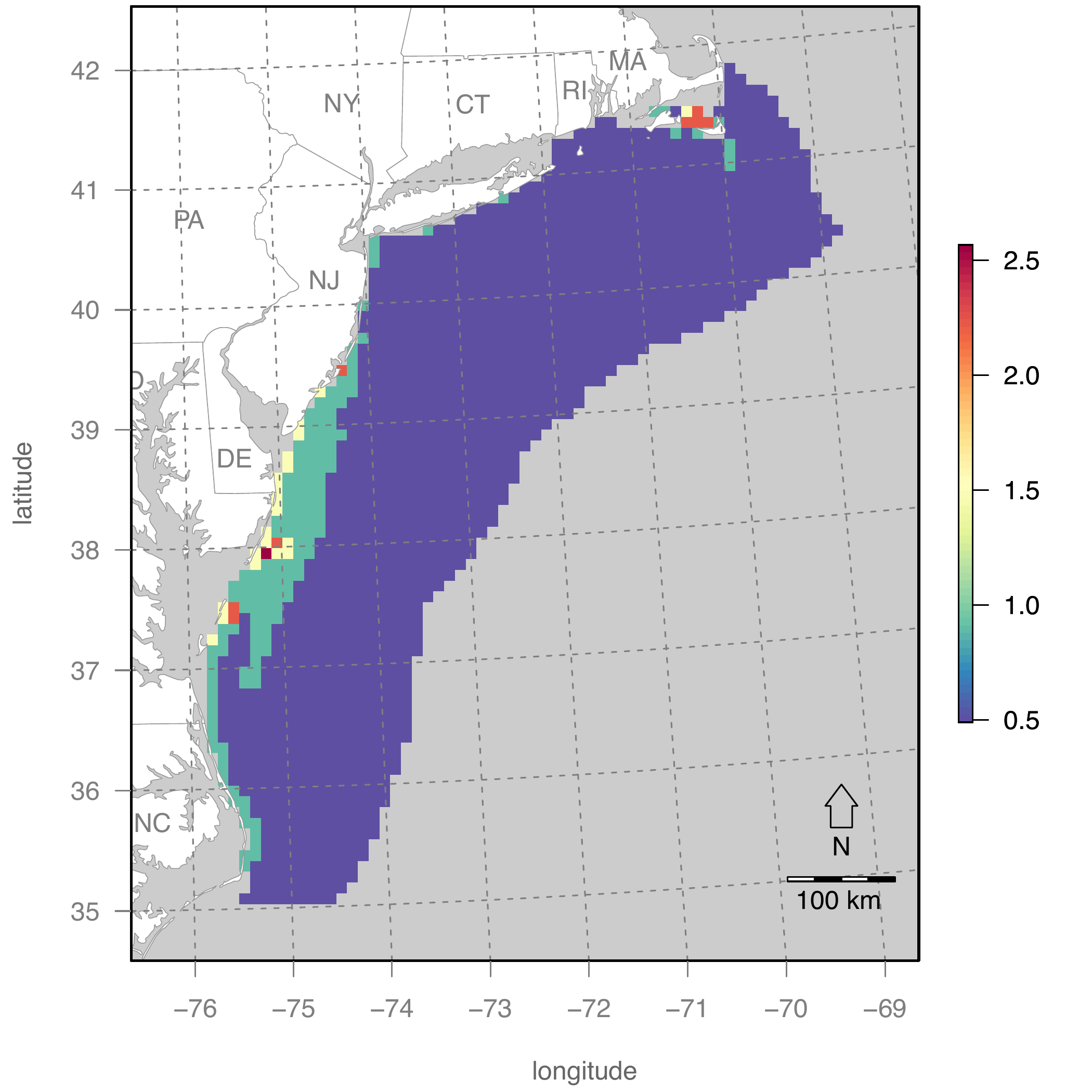

Supplement: S5 Fig — (TIFF) [file pone.0215722.s005.tiff]

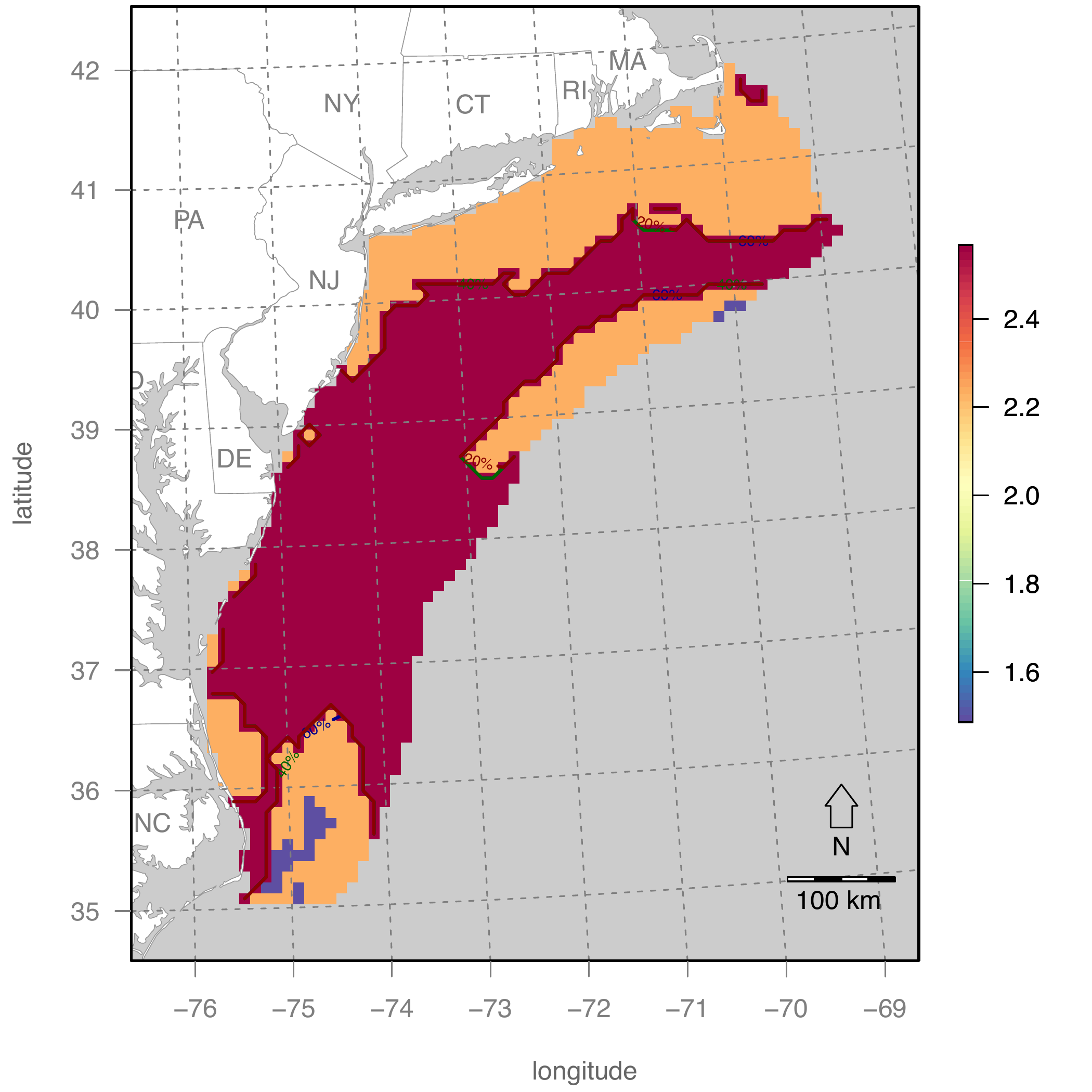

Supplement: S6 Fig — (TIFF) [file pone.0215722.s006.tiff]

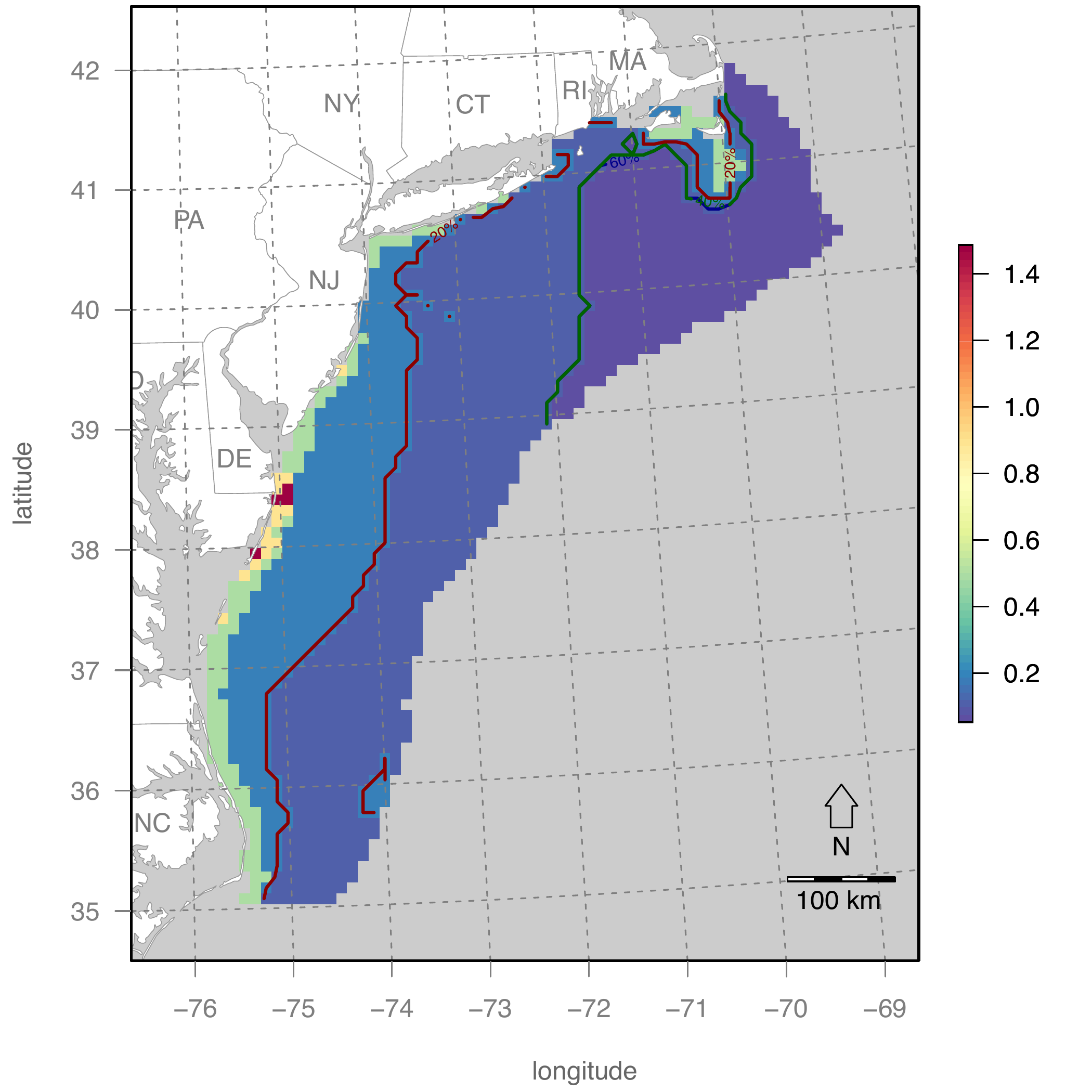

Supplement: S7 Fig — (TIFF) [file pone.0215722.s007.tiff]

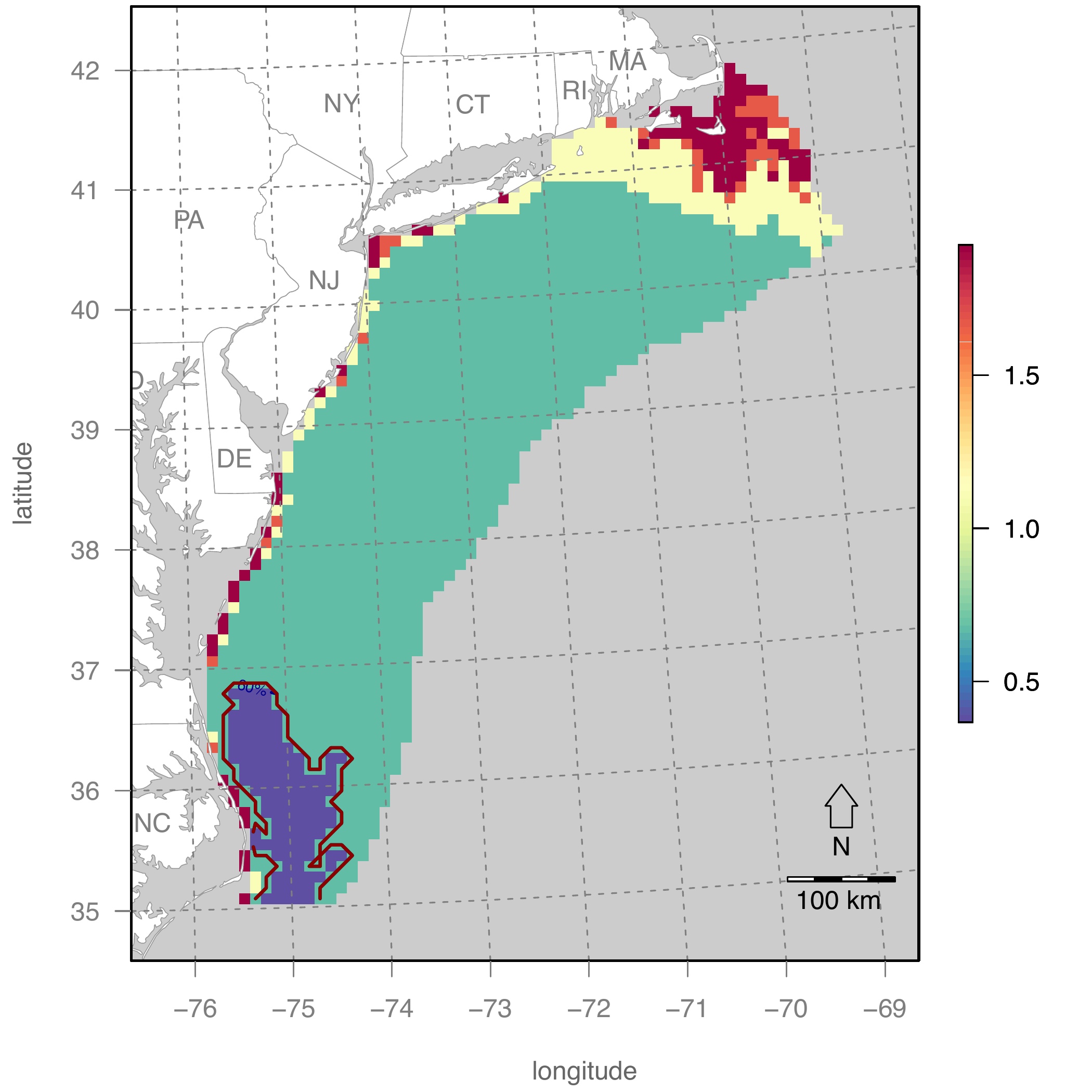

Supplement: S8 Fig — (TIFF) [file pone.0215722.s008.tiff]

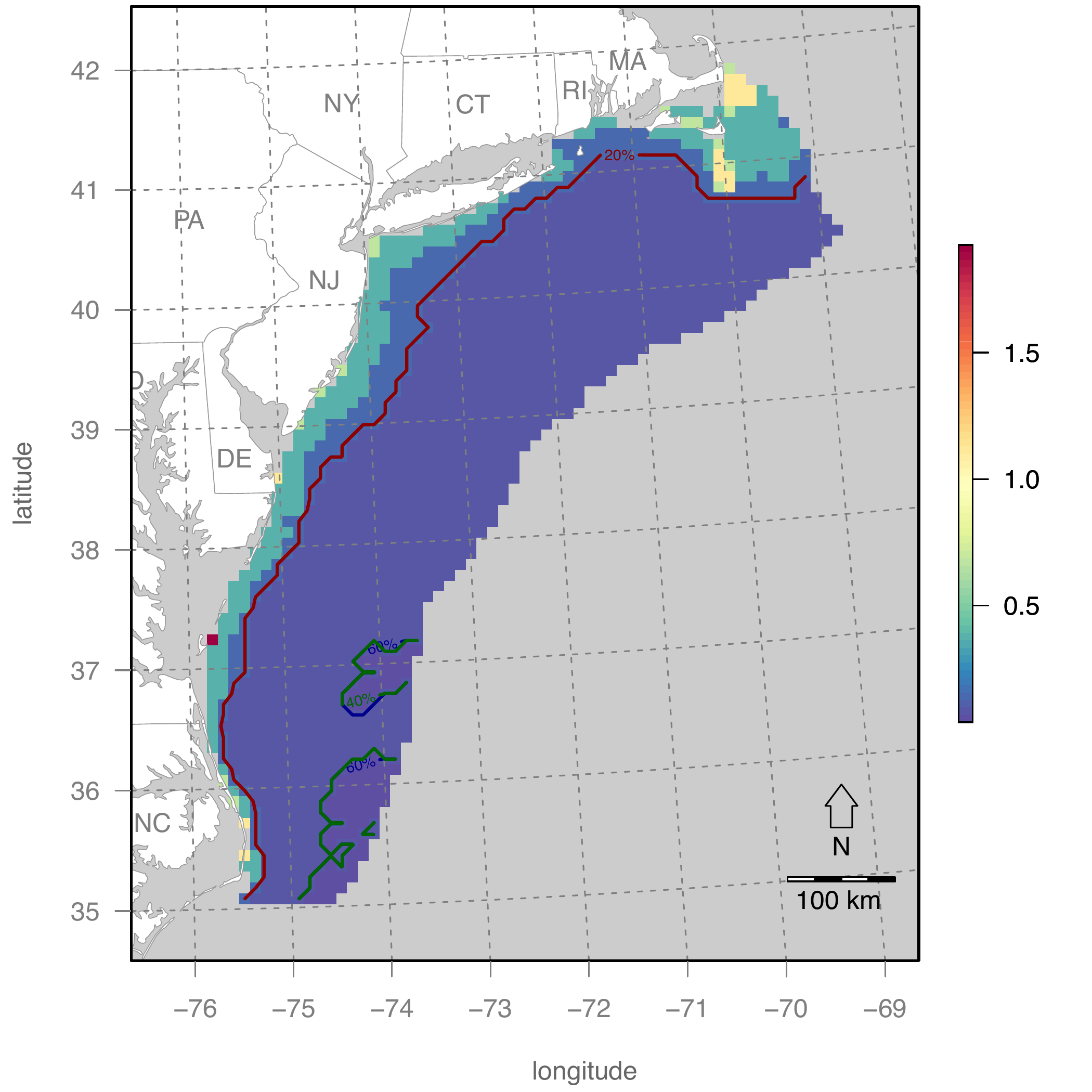

Supplement: S9 Fig — (TIFF) [file pone.0215722.s009.tiff]

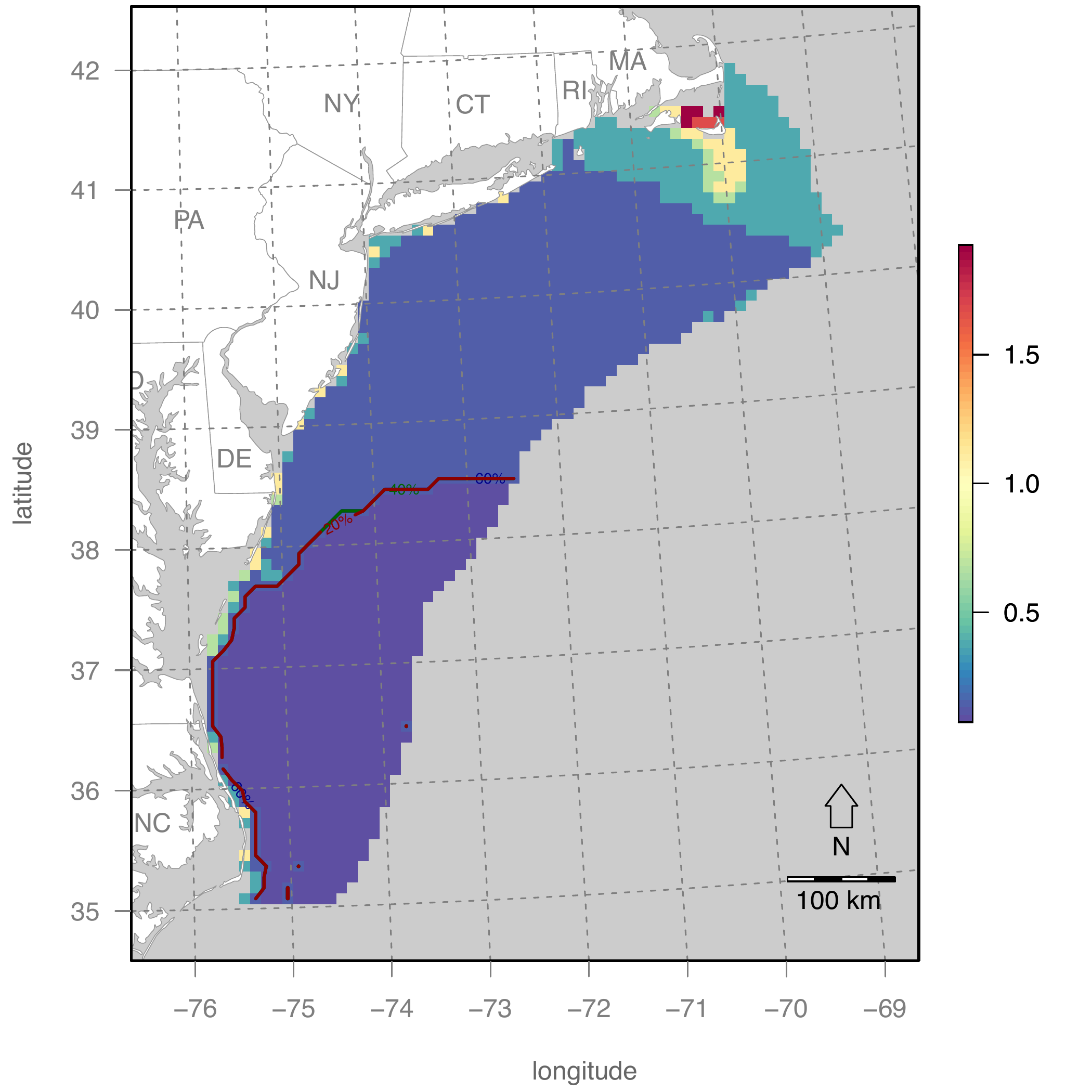

Supplement: S10 Fig — (TIFF) [file pone.0215722.s010.tiff]

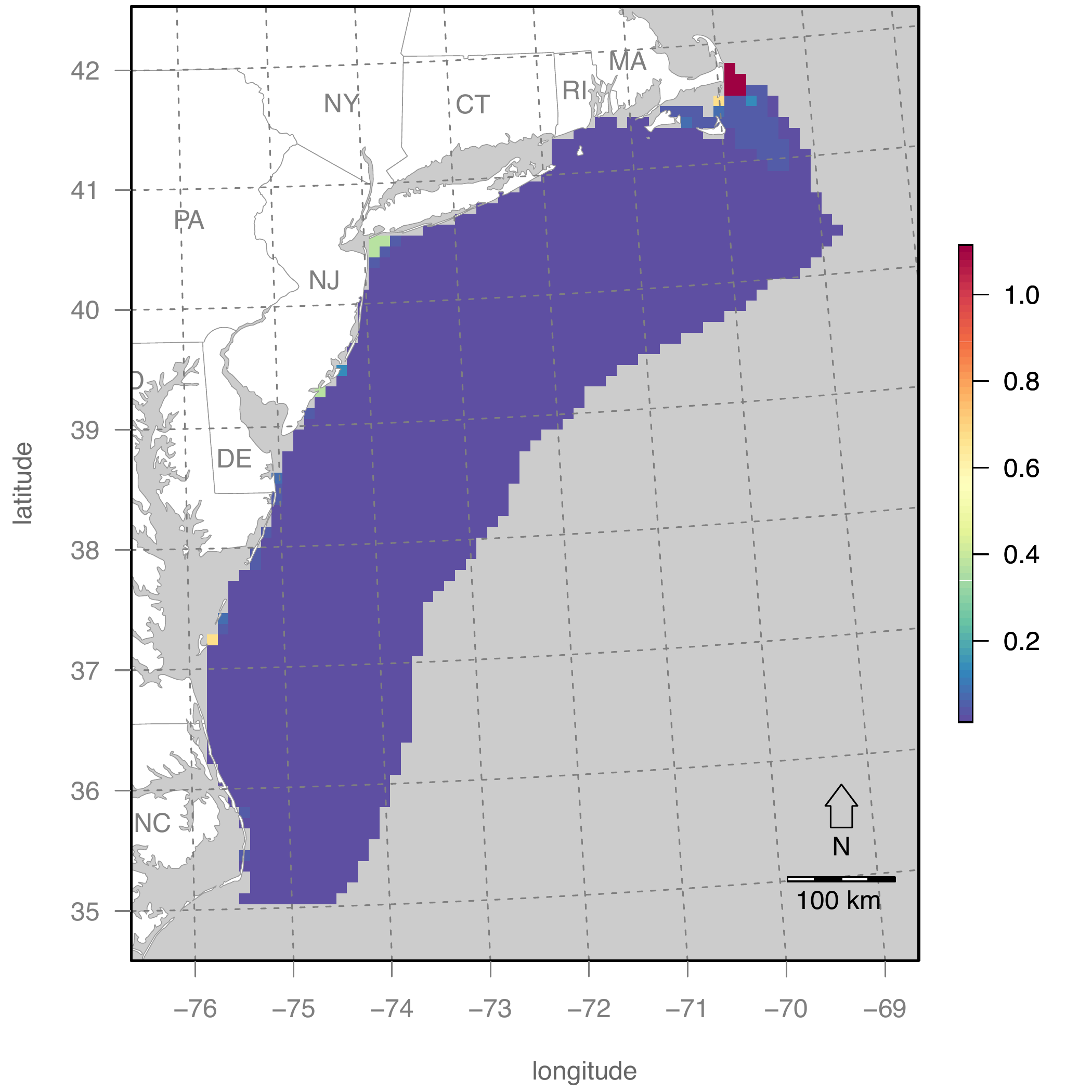

Supplement: S11 Fig — (TIFF) [file pone.0215722.s011.tiff]

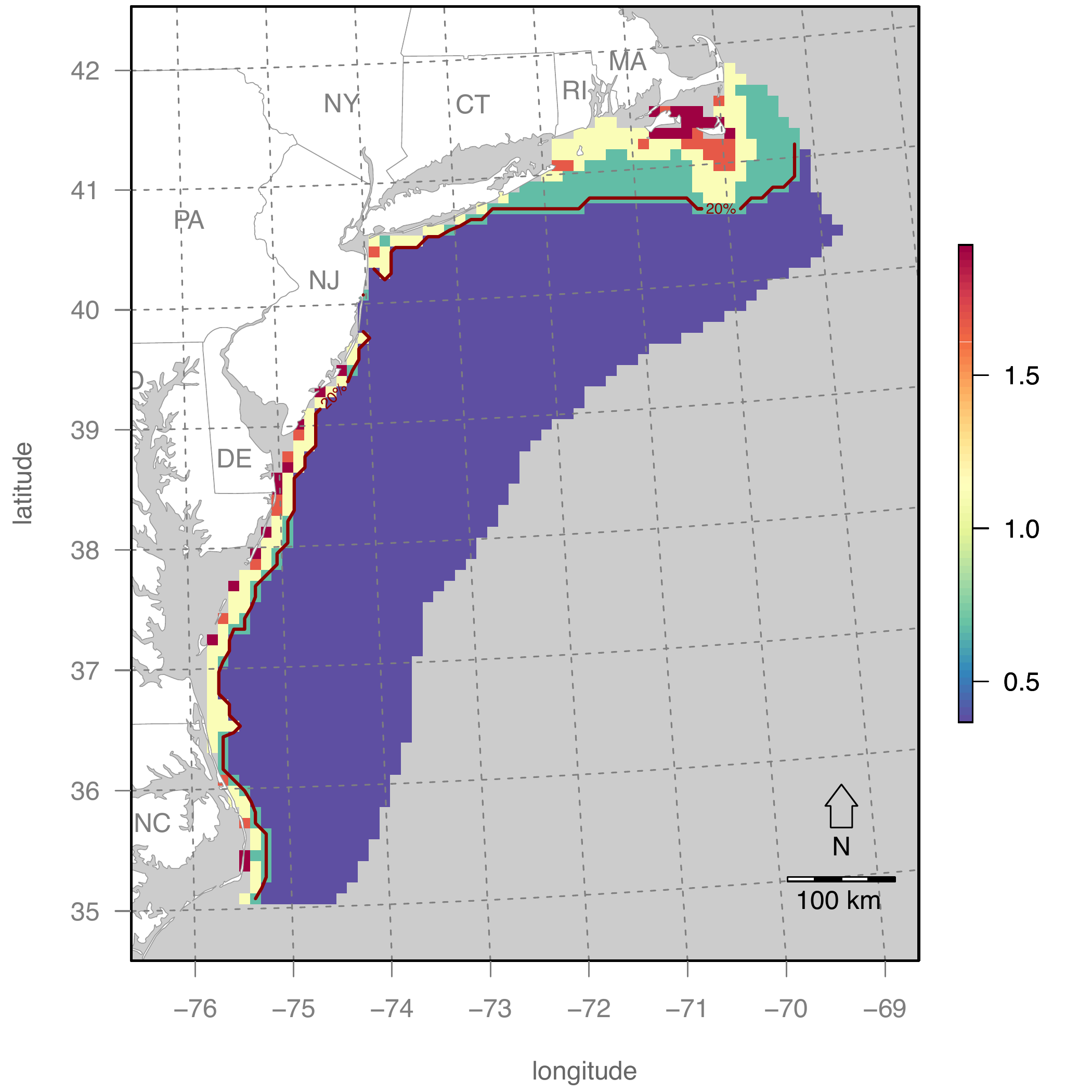

Supplement: S12 Fig — (TIFF) [file pone.0215722.s012.tiff]

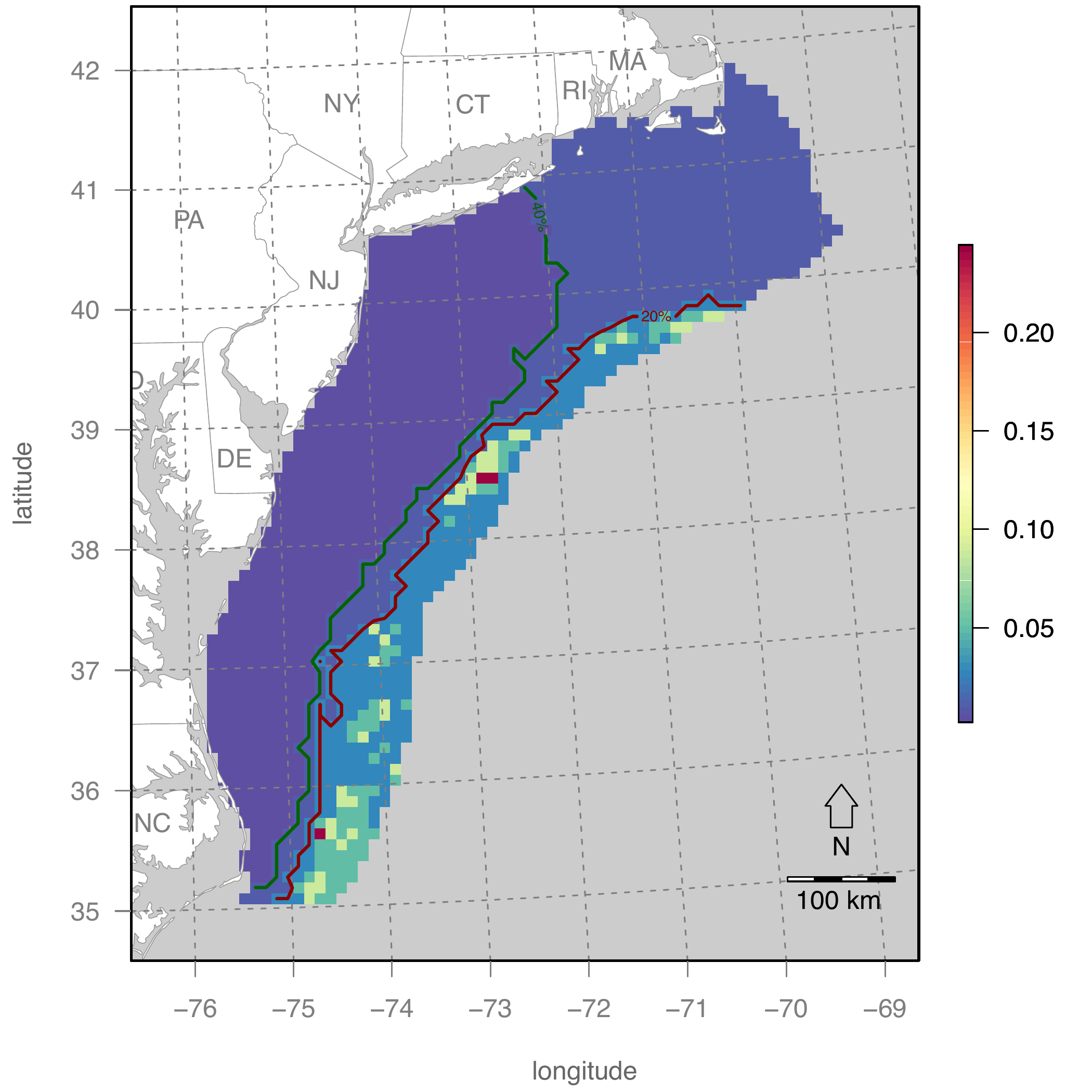

Supplement: S13 Fig — (TIFF) [file pone.0215722.s013.tiff]

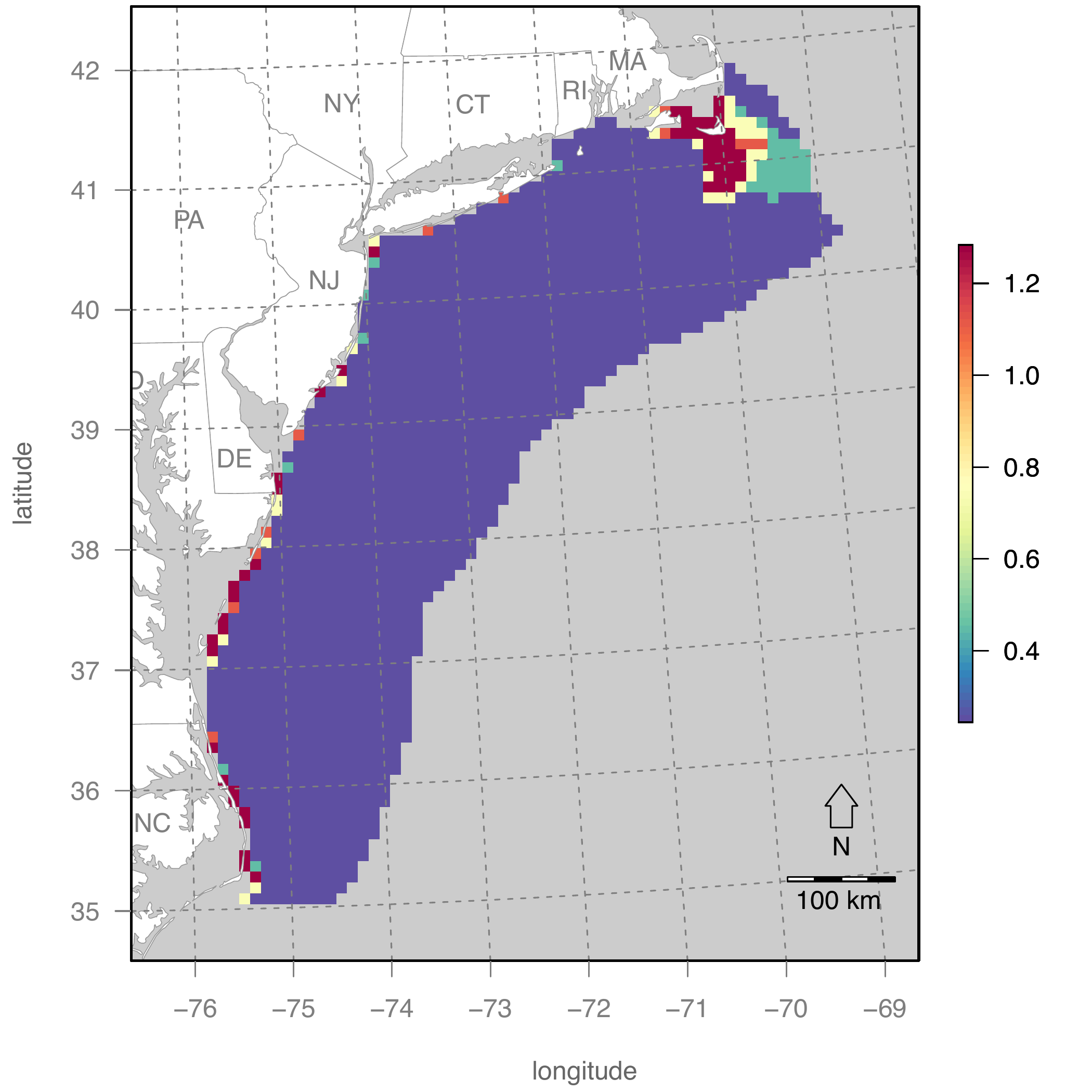

Supplement: S14 Fig — (TIFF) [file pone.0215722.s014.tiff]

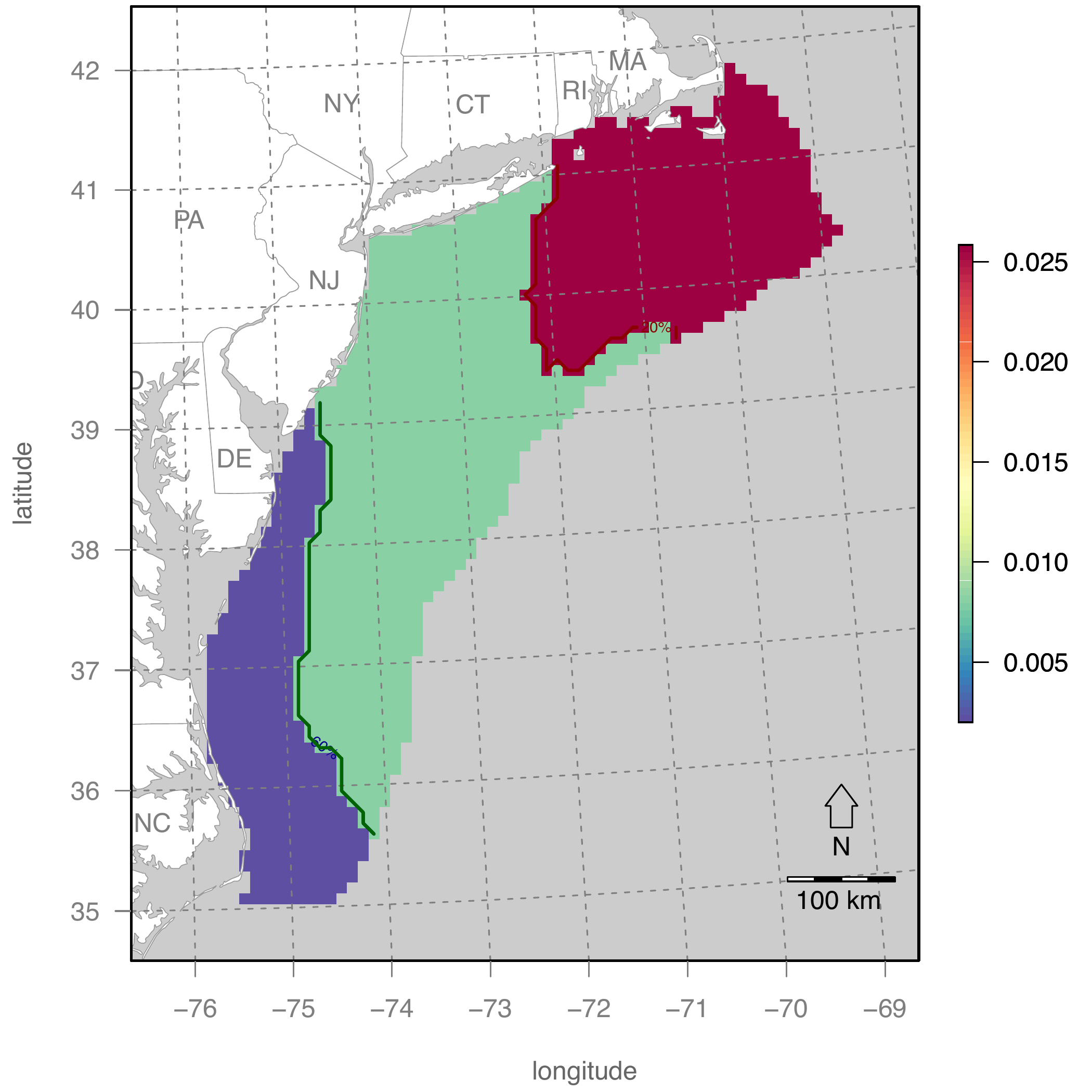

Supplement: S15 Fig — (TIFF) [file pone.0215722.s015.tiff]

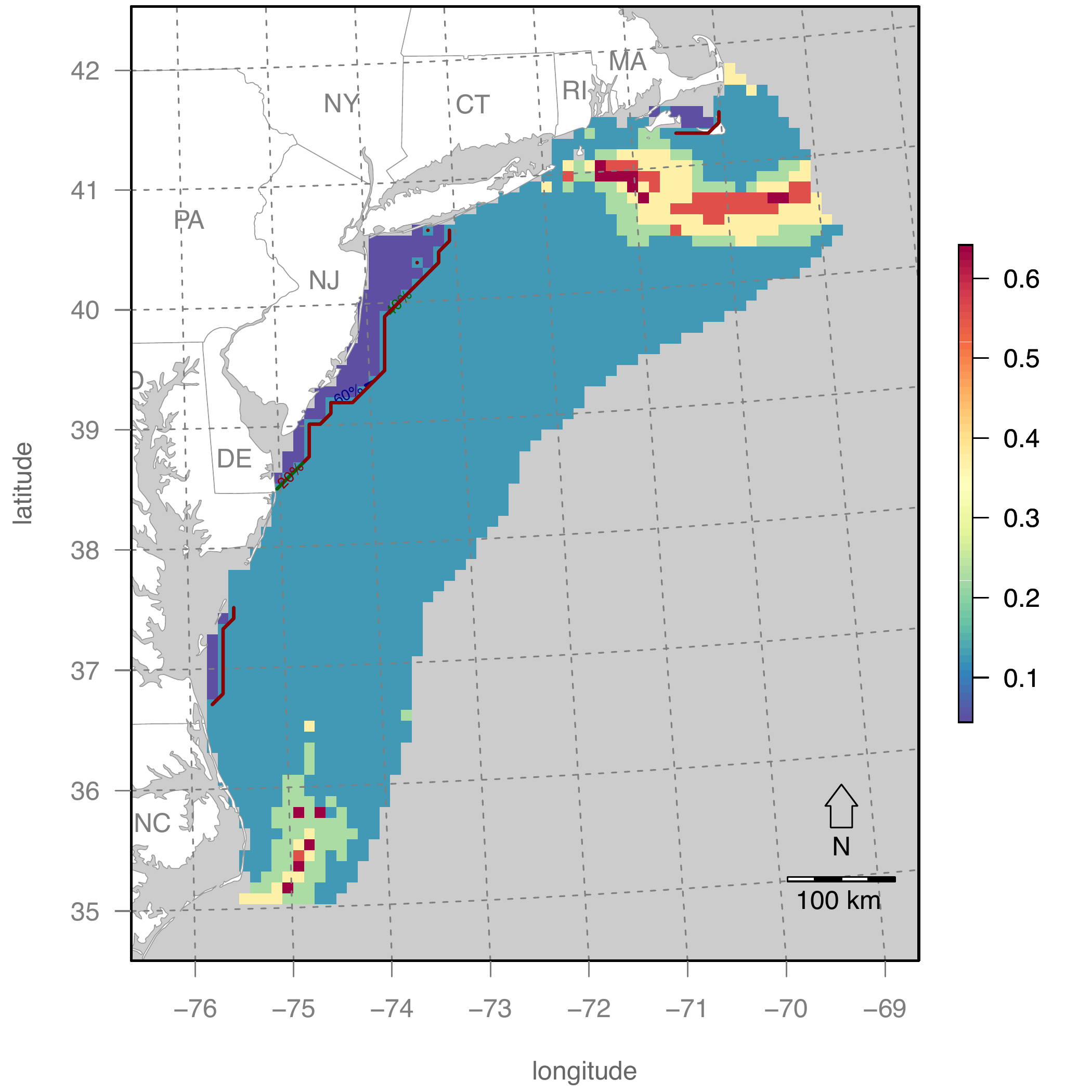

Supplement: S16 Fig — (TIFF) [file pone.0215722.s016.tiff]

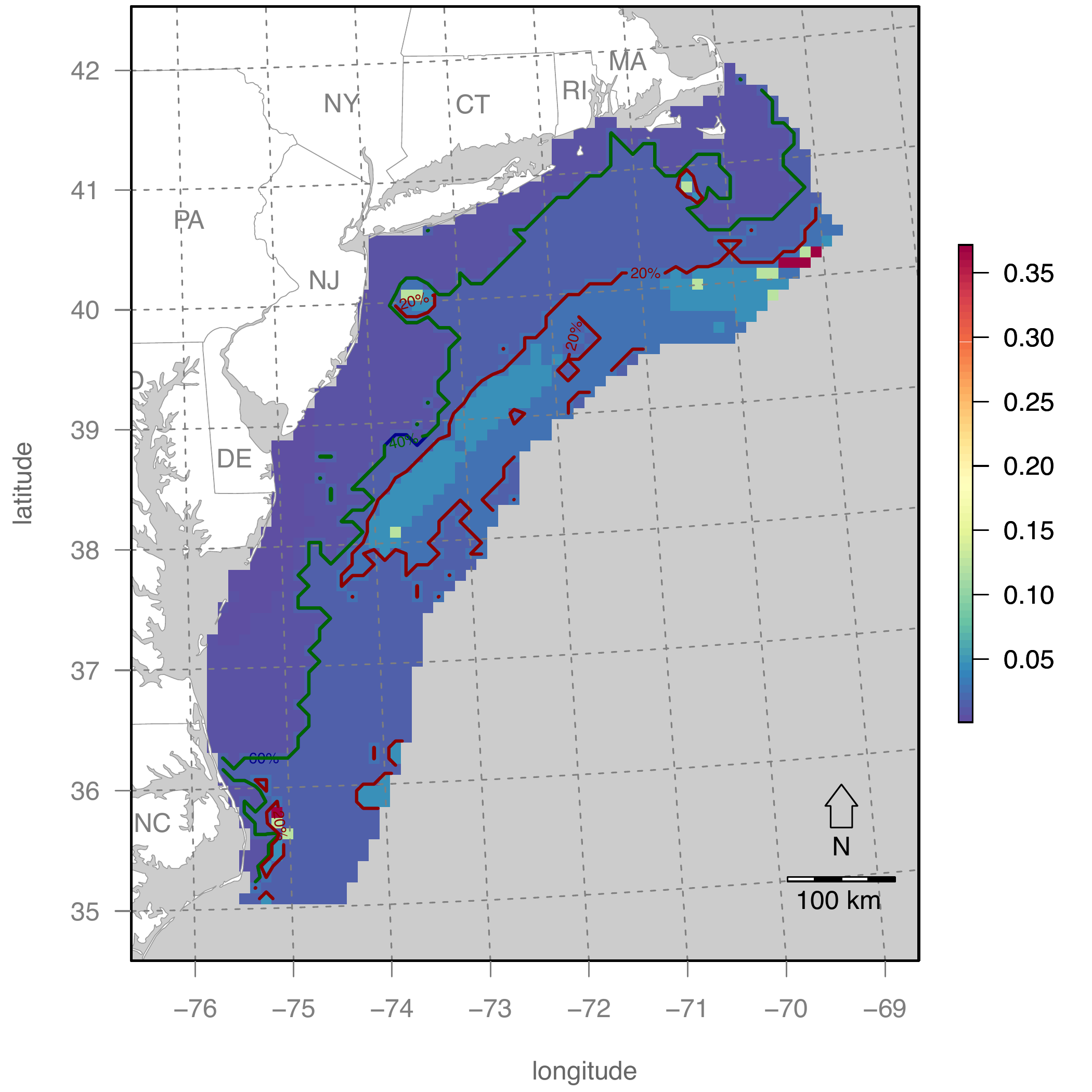

Supplement: S17 Fig — (TIFF) [file pone.0215722.s017.tiff]

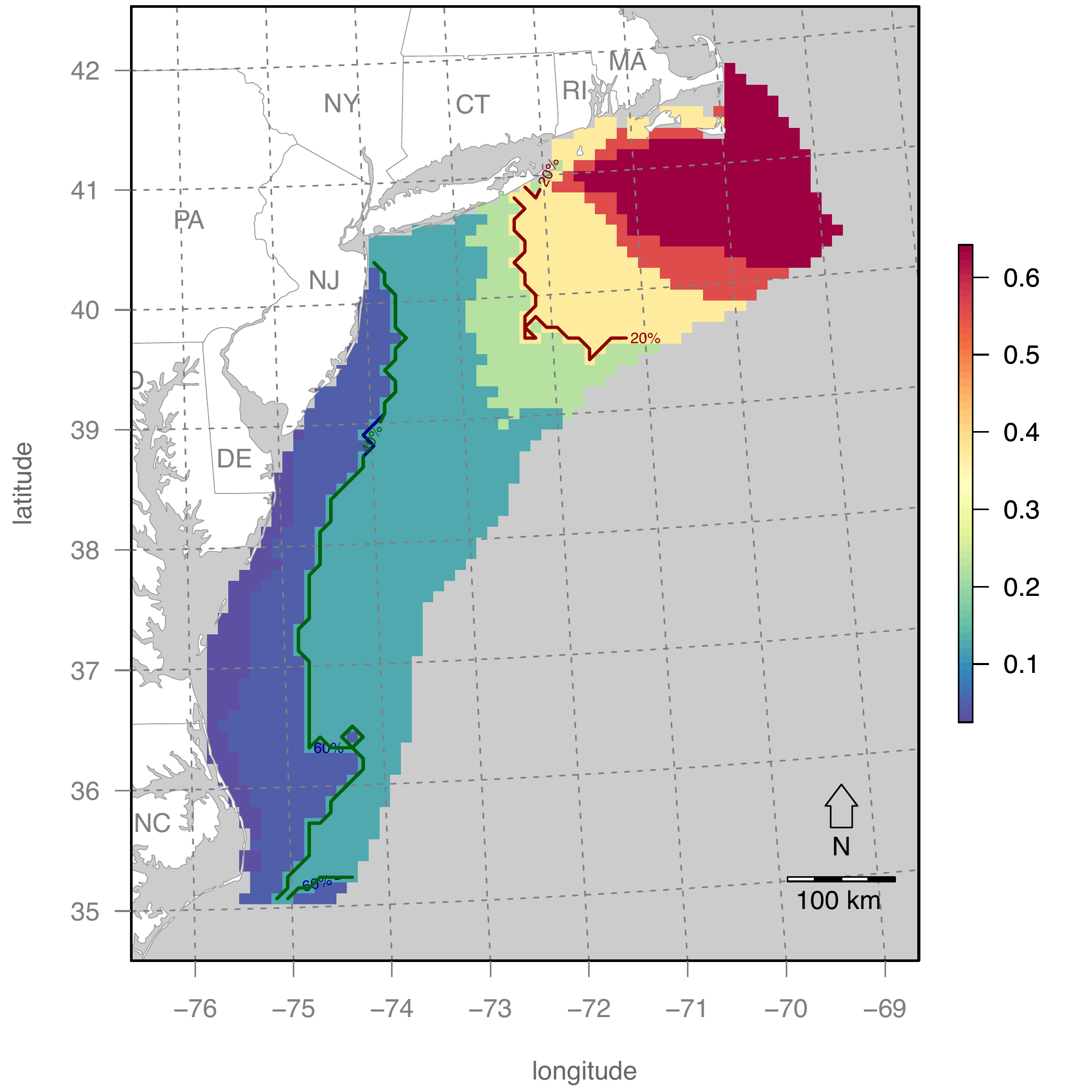

Supplement: S18 Fig — (TIFF) [file pone.0215722.s018.tiff]

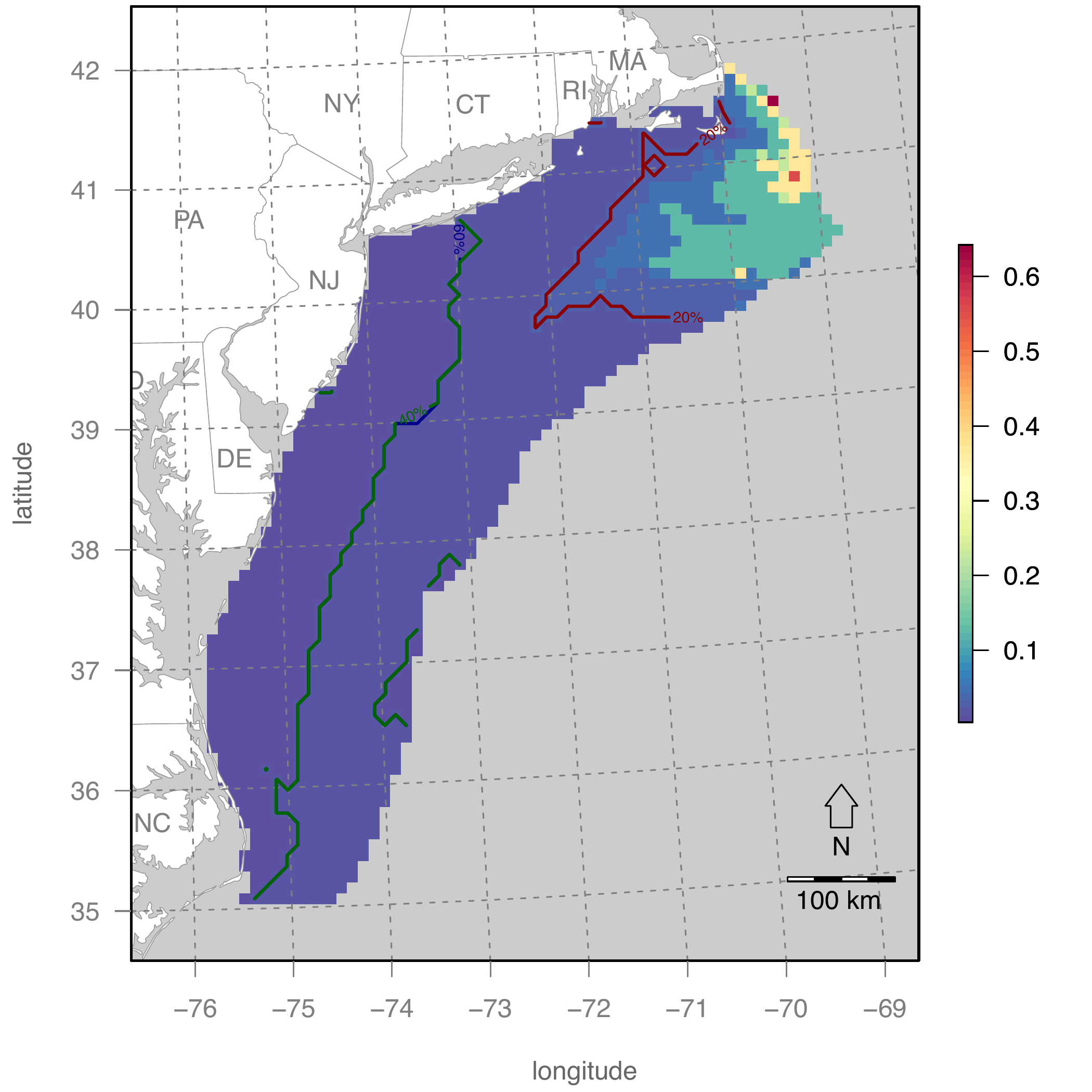

Supplement: S19 Fig — (TIFF) [file pone.0215722.s019.tiff]

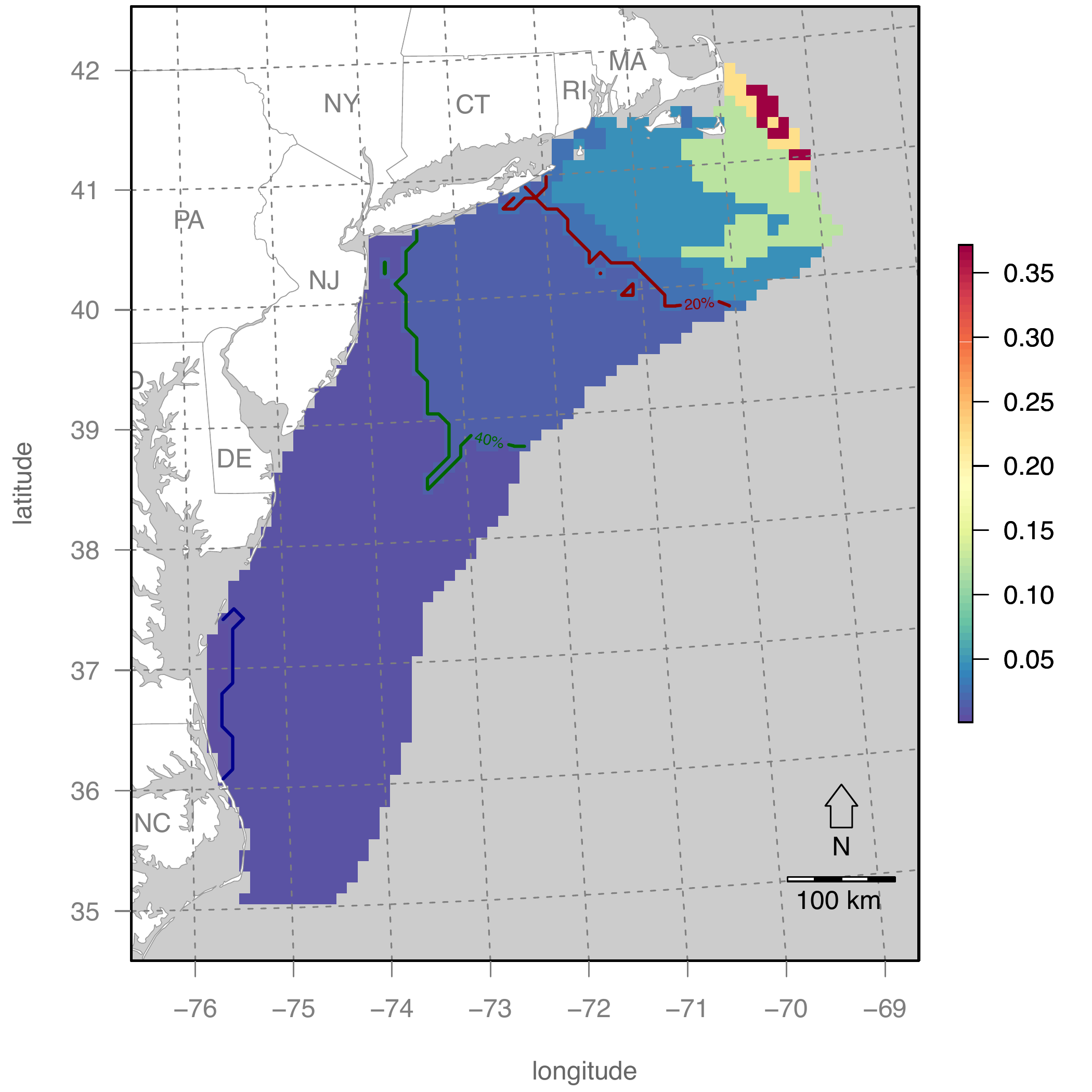

Supplement: S20 Fig — (TIFF) [file pone.0215722.s020.tiff]

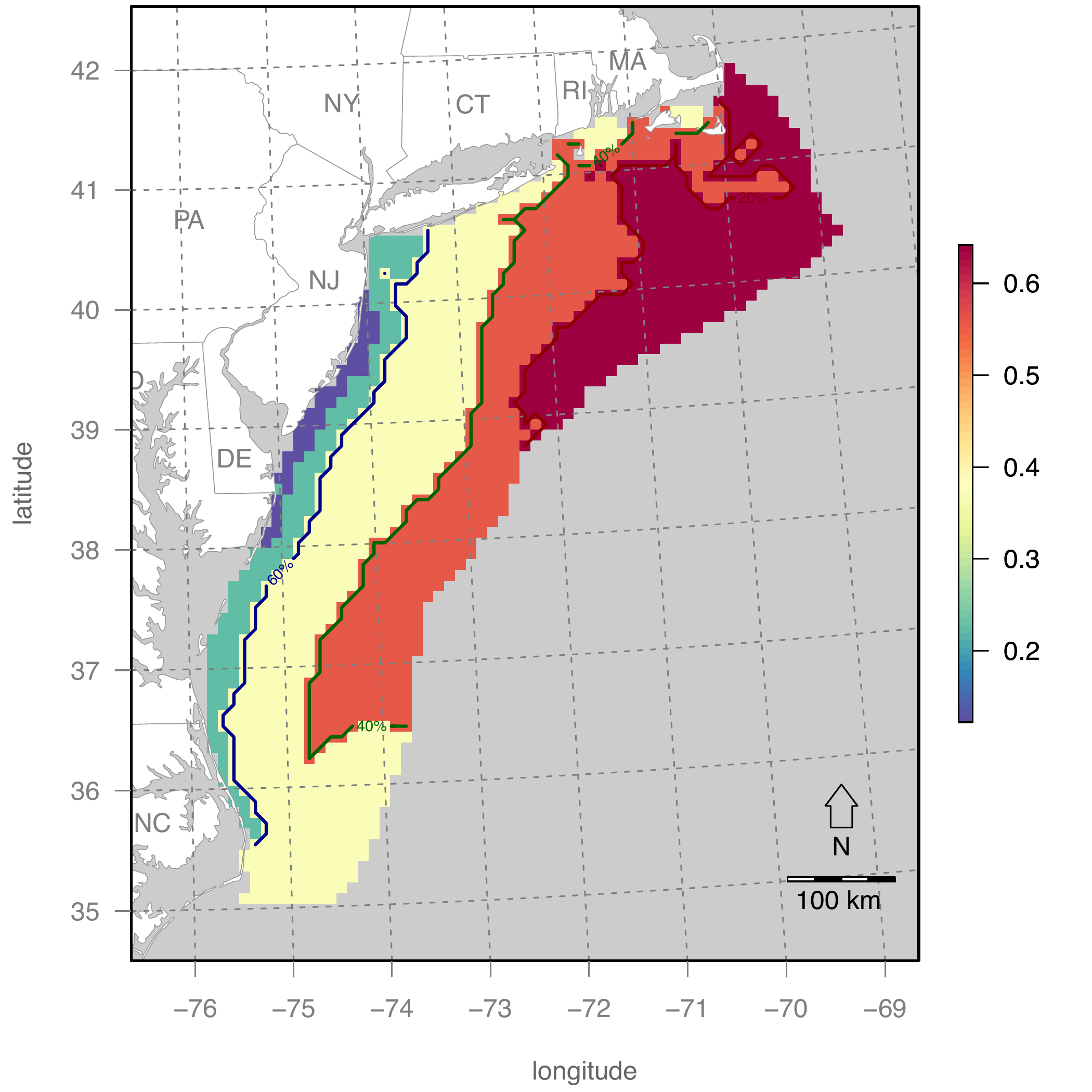

Supplement: S21 Fig — (TIFF) [file pone.0215722.s021.tiff]
